# Sustainable Polymers from Recycled Waste Plastics and Their Virgin Counterparts as Bitumen Modifiers: A Comprehensive Review

**DOI:** 10.3390/polym13193242

**Published:** 2021-09-24

**Authors:** Sabzoi Nizamuddin, Yeong Jia Boom, Filippo Giustozzi

**Affiliations:** Civil and Infrastructure Engineering, School of Engineering, RMIT University, Melbourne, VIC 3001, Australia; nizamuddin.nizamuddin@rmit.edu.au (S.N.); s3359878@student.rmit.edu.au (Y.J.B.)

**Keywords:** recycled plastics, plastomers, asphalt, bitumen, recycling, sustainability

## Abstract

The failure of bituminous pavements takes place due to heavy traffic loads and weather-related conditions, such as moisture, temperature, and UV radiation. To overcome or minimize such failures, a great effort has been put in recent years to enhance the material properties of bitumen, ultimately improving field performance and increasing the pavement service life. Polymer modification is considered one of the most suitable and by far the most popular approach. Elastomers, chemically functionalised thermoplastics and plastomers * (* Note: notwithstanding the fact that in Polymer Science the word ‘plastomer’ indicates a polymer with the simultaneous behaviour of an elastomer and plastics (thermoplastics), this paper uses the term ‘plastomer’ to indicate a thermoplastic polymer as it is more commonly found in Civil and Pavement Engineering.) are the most commonly used polymers for bitumen modification. Plastomers provide several advantages and are commonly acknowledged to improve high-temperature stiffness, although some of them are more prone to phase separation and consequent storage instability. Nowadays, due to the recent push for recycling, many road authorities are looking at the use of recycled plastics in roads. Hence, some of the available plastomers—in pellet, flakes, or powder form—are coming from materials recycling facilities rather than chemical companies. This review article describes the details of using plastomers as bitumen modifiers—with a specific focus on recycled plastics—and how these can potentially be used to enhance bitumen performance and the road durability. Chemical modifiers for improving the compatibility between plastomers and bitumen are also addressed in this review. Plastomers, either individual or in combination of two or three polymers, are found to offer great stiffness at high temperature. Different polymers including HDPE, LDPE, LLDPE, MDPE, PP, PS, PET, EMA, and EVA have been successfully employed for bitumen modification. However, each of them has its own merit and demerit as thoroughly discussed in the paper. The recent push in using recycled materials in roads has brought new light to the use of virgin and recycled plastomers for bitumen modification as a low-cost and somehow environmental beneficial solution for roads and pavements.

## 1. Introduction

Bitumen (or asphalt binder) is a by-product of the petroleum industry obtained by distillation of crude oil. It possesses valuable characteristics including long durability, high adhesion and water proofing abilities, which opened its ways for utilization as a road construction material [1]. In road construction, the bitumen is mixed with aggregates as a binder for producing the asphalt mixture. The performance characteristics and overall durability of asphalt mixtures highly depend upon the performance of the bitumen binder. Failure of asphalt pavements is mostly directly related to the failure of the asphalt binder, which takes place due to either thermal cracking occurring at low temperature, rutting at high temperature resulting in softening of the bitumen and reduced elasticity of the bitumen, or due to fatigue cracking at intermediate temperature caused by cyclic loads and ageing of the pavement [1]. 

**Figure 1:**
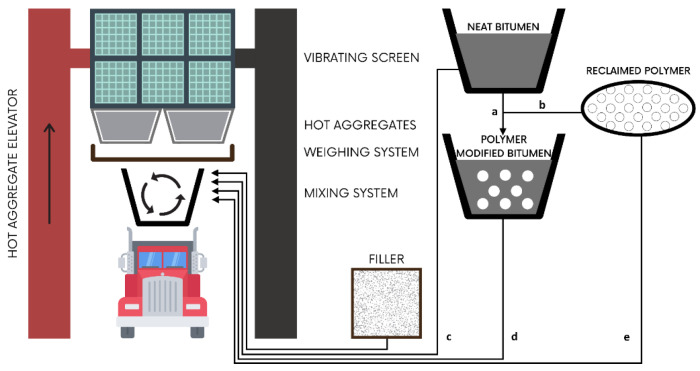
Sketch of the dry mixing process (when lines a, b, d are closed and lines c and e are opened) and the wet mixing process (when lines a, b, d are opened and lines c and e are closed) for mixing polymers with bitumen [12].

As maintenance and repair of bitumen pavements are undesirable for socio-environmental and economic reasons, considerable efforts are spent to avoid failures [2]. A large amount of studies focused on investigating the modification of bitumen to obtain enhanced durability and high-quality pavements. Among all investigated modification methods, polymer modification of bitumen is considered one of the most suitable and by far the most popular approach [3]. The polymer is incorporated into the bitumen either by chemical reaction (wet process) or mechanical mixing (dry process) to get polymer-modified bitumen [4,5]—as shown in Figure 1. In the wet process, the polymer and bitumen are directly blended at high temperature for a given time to allow for proper chemical and physical interaction between the constituents. When the polymer is refined from waste plastics, for instance, the wet method incorporates plastic in the form of flakes, pellets, or powder into hot bitumen [6]. With common mixing temperatures of 160–170 °C [7,8,9], plastics selected for the wet method generally require melting temperatures below the specified range. Generally, polymer modification of bitumen through the wet process enables improvements in the areas of elasticity, adhesion, cohesion, and stiffness, ultimately resulting in higher durability, fatigue life, and resistance to rutting [10,11].

In the dry process, the polymer and bitumen are not premixed, and the polymer is added directly into the aggregates at the beginning of the mixing process [12,13]. When using waste plastics in the dry method, recycled plastic is incorporated into the bitumen mix as a substitute of the aggregate and it is suggested that its melting temperature is above the bitumen mixing temperature [14,15]; however, several studies have used low-temperature melting point plastics with the dry method as a pre-coating of the hot aggregate before adding the bitumen. The latter is sometimes known as the ‘mixed’ method. The dry method allows using a greater percentage of plastics as it reduces the amount of aggregates to be used in the total mixture. Studies have shown that by adopting this method, stiffness, fatigue life, and Marshall stability characteristics of the road pavement mix are improved [10,11,16,17]. However, several shortcomings have also been identified for both methods (wet and dry) as further described in the following sections. 

The blending/mixing process—either chemical or mechanical—has a significant effect on the total cost of the operation and overall properties of the final blend [18,19]. For instance, the resulting blend may experience primary ageing due to the higher blending temperature involved, where degradation of the polymer and oxidation of the maltene compounds in bitumen (i.e., the low molecular weight compounds) can occur [20,21]. In addition, the difference in polarity and molecular weight of the bitumen and polymer affects the compatibility of the two phases. Further, polymer-modified bitumen blends are not always thermodynamically stable and are prone to phase separation during storage at high temperature [22,23,24,25]. 

Together with the benefits of polymer-modified bitumen, there are some limitations and challenges which need to be taken into consideration such as the high cost of virgin polymers, resistance to ageing, sensitivity to high temperatures for some types of polymer modified bitumen (i.e., waxes-modified bitumen [26]), low elasticity, and poor storage stability [3]. To come up with these shortcomings, several techniques and methods have been suggested such as sulphur vulcanization, saturation, addition of antioxidants, functionalization, application of reactive polymers, and utilization of hydrophobic clay minerals [3,27].

Several review articles have been published on polymer modification of bitumen; some of them focused on the effect of mixing conditions and resulting in modified bitumen properties, whereas others focused on elastomers—the most adopted polymers in the road sector—and reactive polymer applications. However, to the authors’ best knowledge, there is minimal comprehensive information published on the utilization and comparison between waste (recycled plastics) and virgin plastomers for road applications. Therefore, the main objective of this paper is to provide an extensive overview on waste (i.e., coming from recycling operations) and virgin plastomer modification of bitumen for pavement applications. The background and physio-chemical characteristics of bitumen are briefly discussed in the following section. Further, the incorporation methods of polymer with bitumen are evaluated. Different characteristics of plastomer-modified bitumen including chemical, thermal, rheological, structural, and mechanical properties are investigated. A description of the processes that lead to the manufacturing of recycled plastics is also provided. Finally, a critical discussion about plastomer-modified bitumen together with conclusions and recommendations for future research work are identified.

A systematic literature review methodology was adopted in this study to systematically review and collect a wide amount of the literature reported in the last decade (2010–2020); however, some of the important literature studies before 2010 are also cited in this study. To conduct the literature survey, three research databases including Scopus, Web of Science, and Google Scholar were used. The keywords used for searching through the literature include ‘bitumen’, ‘bitumen modification’, ‘polymer modified bitumen’, ‘PMB’, ‘virgin and recycled polymers used for bitumen modification’, ‘performance of PMB’, ‘hybrid polymer’, and ‘chemical modifiers’, among the most relevant.

## 2. Bitumen 

Bitumen is a well-known engineering material and is obtained from the fractional distillation of crude petroleum oil. Although the chemical composition of bitumen is variable and complex, commonly, it is divided into four general fractions including aromatic hydrocarbons (80% carbon, 15% hydrogen [28,29]), asphaltenes, resins, and saturates [1]. Generally, bitumen consists of 11.9–15.8% saturates, 39.6–53.1% aromatics, 22.8–34.8% resins, and 10.3–12.1% asphaltenes (Table 1) depending on the country of origin and refinery process [3]. The reported characteristics of standard paving bitumen in different studies—although these figures may be different worldwide depending on the bitumen source—are shown in Table 1. SARA (Saturate, Aromatic, Resin, and Asphaltene) composition and other basic characteristics of standard bitumen are also listed in Table 1. SARA composition of bitumen can broadly be divided into asphaltenes (q black coloured part of bitumen which is insoluble in n-heptane) and maltenes (the combination of resins, aromatics, and saturated compounds which are soluble in n-heptane) [30]. 

Due to its properties, bitumen has been used for different applications including adhesives, preservatives, sealants, water proofing agents, and as a construction material for roads and airports [31]. It is reported that 85% of the total consumption of bitumen is used to build pavements of different nature [32] although—in its standard unmodified state—it is still facing challenges due to the lack of suitable mechanical properties in certain environments, mainly caused by its thermal susceptibility [33]. This suggests adopting techniques for enhancing bitumen performance, such as polymer modification [34]. Synthetic polymer modification of neat bitumen provides significant improvements on a wide range of bitumen properties, enhancing the demand for polymer-modified bitumen [35]. The most commonly used polymers for the modification of bitumen include approximately 75% of elastomers, 15% plastomers, and 10% shredded vehicle tyre rubber and other types of materials [36]. 

**Table 1:** General characteristics of standard paving bitumen (unmodified).

Property	Value	References
Density (g/cm^3^)	1.004–1.019	[37,38,39,40]
Penetration (0.1 mm)	59.10–98.0	[37,41,42,43]
Penetration index (PI)	0.152–0.601	[44,45]
Softening point (°C)	42–65	[37,38,39,41,42,44,46,47]
Flash Point (°C)	240–350	[37,41,42]
Fire Point (°C)	270–376	[42]
Ductility (mm)	76–720	[37,41,42,46]
Viscosity @ 135 °C (cP)	100–460.35	[37,41]
Saturates (%)	4.0–15.8	[7,40]
Aromatics (%)	39.6–69.0	
Resins (%)	15.0–34.8	
Asphaltenes (%)	9.0–14.0	
Colloidal index	0.190–0.333	[7]

## 3. Modification of Bitumen 

The concept of mixing two or more materials—with completely different characteristics than those of the parent materials—to form a new product for paving applications has been in practice since the last few decades [48]. The resultant phase behaviour in terms of homogeneity is considered to determine the chemical, electrical, mechanical, rheological, and other characteristics of the product. The homogeneity/miscibility of mixtures depends upon entropy and heat of mixing. The miscibility or homogeneity of polymers in bitumen can be enhanced by adding compatibilizers, cross-linking agents, and by controlling the phase morphology during the blending process [49]. 

It is recommended that the substances used for modification of bitumen should possess the following characteristics: (i) not deteriorate at the production temperature of bitumen mixtures, (ii) retain good chemical compatibility with bitumen, (iii) increase the resistance to deformation and reduce thermal susceptibility, and (iv) being physically and chemically stable by means of not changing their characteristics during transportation, storage, processing, and other operations [50]. Polymer additives enhance bitumen’s mechanical properties, improve bitumen-aggregate adhesion, and reduce temperature susceptibility, which in turn improves the overall performance of the bitumen mixture, stiffness at high temperature, moisture resistance, enhanced fatigue life and resistance to cracking at low temperature [51,52,53,54,55]. However, the final properties of polymer-modified bitumen highly depend upon the singular properties of the polymer and bitumen, the dosage and type of polymer added to the bitumen as well as the blending process [48]. The reactivity and chemical structure of some polymers also affect the compatibility with bitumen, which is directly related to the properties of polymer-modified bitumen [56]. The compatibility of bitumen with polymer modifiers is controlled by various properties of both the polymer and bitumen. When polymers are blended with bitumen, phase separation can occur due to the high molecular weight of polymers as well as inadequate maltene fractions for solvation. Phase separation causes the formation of heterogeneous mixture, resulting in poor storage stability and poor compatibility between the polymer and bitumen [57]. These properties include molecular weight, density, solubility, and polarity, among others [58]. The compatibility between polymers and bitumen is reflected in the storage stability of the polymer-modified bitumen, with better polymer-bitumen compatibility generating greater storage stability and easier handling at the bitumen plant [3]. 

Although polymers have the ability of improving bitumen performance, the blending of bitumen and polymers still poses some challenges due to higher processing temperature and dedicated facilities—hence increased costs at the plant and phase separation due to poor polymer-bitumen compatibility and, sometimes, to the high polymer content. It is suggested that a bitumen modifier should retain the following characteristics: (i) highly soluble into bituminous mixtures to generate a viscous mixture that stays homogenous during storage; (ii) highly resistant to water, thermal stresses, and ultraviolet radiation, (iii) should not release dangerous substances to the environment and (iv) be widely available [22,29,59]. 

Both virgin and, more recently, recycled polymers have been successfully employed for bitumen modification. Evidently, the research for utilizing recycled plastics in the bitumen industry has significantly increased in the past decade because of the recycling push many countries are putting in place due to growing waste issues such as disposal, environmental and health concerns of plastic wastes. Waste plastics disposal is one of the main growing issues around the world due to rapid increase in plastics consumption rate. The environmental impacts include entrapment and destruction of habitats for wildlife, hazard of ingestion, plastic-facilitated transportation of organisms to eco-system, whereas other health concerns include circulatory, respiratory, and lymphatic system problems for transport with ultimate deposition in kidney, gut, and liver. To minimize the health and environmental issues due to this waste while still improving bitumen performance, there is now great attention to use waste-recycled plastic polymers in roads [60]. It should be noted that research on waste plastics in bitumen started approximately 20 years ago although it did not reach much attention until recently, as governments are heavily investing in recycling and green technology. Research studies [10,16,61] using waste plastics in bitumen roads have found noticeable improvements in tensile strength, water resistance, durability, and overall service life. In addition, utilizing plastics in the bitumen industry, recycling and use of eco-friendly methods to construct road pavements have projected a potential reduction in carbon emission by one third [62,63]. A more recent LCA (life cycle assessment) study found that recycled plastics in bitumen can be beneficial although this depends on the methodology adopted (wet or dry), with more environmental benefits associated with the wet methodology [64]. 

The polymers used as a bitumen modifier belong to three categories according to their chemical structures and properties [65]; these include plastomers, elastomers, and chemically functionalised thermoplastics [66]. Although it is a fact that polymer modifiers enhance the resistance against thermal susceptibility of bitumen, each type of polymer has a specific effect on bitumen properties [12]. For instance, reactive and plastomer polymers tend to increase the stiffness and resistance to deformation due to load, whereas elastomer polymers improve the elastic properties (resistance to fatigue) of bitumen [67]. It is reported that reactive polymers improve the compatibility between polymer and bitumen as well as require less additives for the stabilisation of the polymer phase. Reactive polymers commonly cause an improvement in the mechanical characteristics, temperature susceptibility and storage stability of the modified binder [68]. Navarro et al. [68] investigated a comparative analysis between reactive and non-reactive polymers on rheological properties of modified binders. They found that reactive polymers caused an evolution in rheological properties (G’ and G”), especially at intermediate temperature and low frequency. In addition, the reactive polymer modified binder remains homogenous, consequently offering better storage stability. Among all three types of polymers used for bitumen modification, plastomers are commonly cheaper and offer high stiffness at high temperatures, and hence, resistance to permanent deformation. Additionally, the melting point temperature of common plastomers is lower than the temperatures used to prepare hot bitumen mixes. Recent research has focussed on recycled plastomers over virgin plastomers for their utilization in road; therefore, the following section expands on recycled plastic waste as possible modification for bitumen. 

## 4. Recycled Plastic Waste and Their Use in Roads 

**Figure 2:**
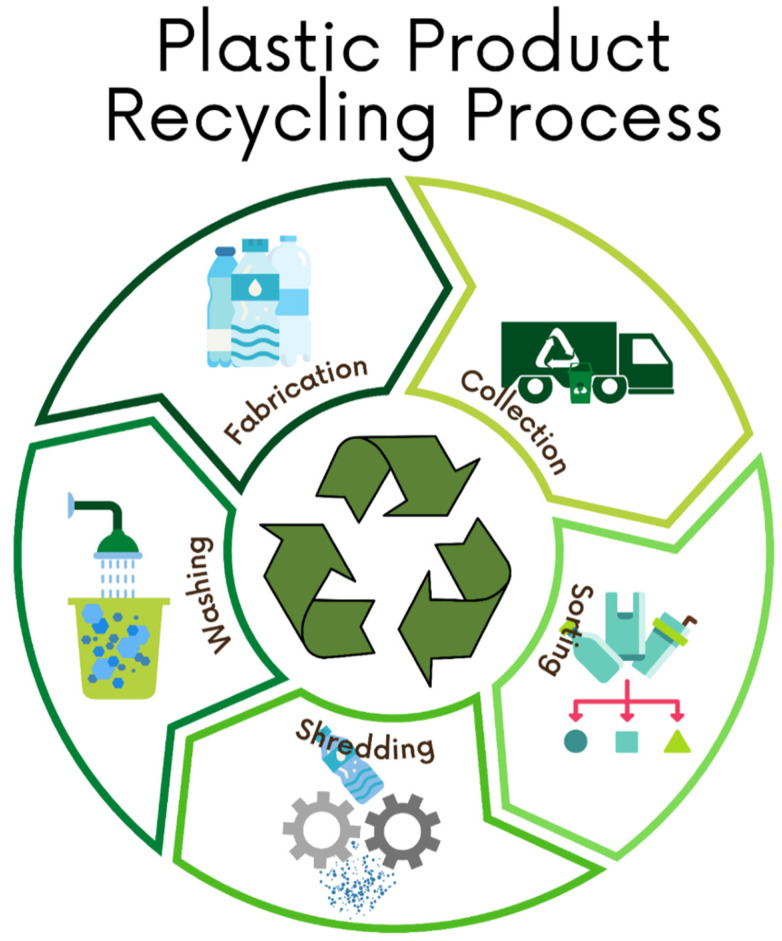
Overall mechanical recycling process of waste plastics.

Plastic consumption around the world has been increasing significantly over the last decades [69,70] causing severe environmental pollution with no alternative ways to dispose and recycle [71,72,73]. In 2017, Victoria in Australia generated 586,300 tons of plastics—only 130,000 tons (22.2%) were recycled while 7200 tons (1.2%) were combusted for energy recovery and the remaining 449,100 tons (76.6%) were sent to landfill. The rate of plastic generation in Victoria was projected to increase by 100,000 tons every 4 years [74]. The US Environmental Protection Agency (EPA) reported that 34.5 million tons of plastics were generated in the United States in 2015. Despite having a recycling rate of 75% from the local citizens, only 3.14 million tons (9.1%) were recycled while 5.35 million tons (15.5%) of plastics were combusted for energy recovery and 26.01 million tons (75.4%) of plastics were sent to landfill. The recycling of polymers and plastic wastes is suggested as a better solution compared to other means of dealing with them such as composting, incineration, or landfilling. Polymers are recycled by two means, i.e., mechanical recycling and chemical recycling. Mechanical recycling is a method of repurposing unmodified plastics into new products [75]. Mechanical recycling comprises of different steps including collection, sorting, shredding, washing or decontamination, extrusion, quenching, and pelletizing (Figure 2). Alternatively, chemical recycling is a method to convert waste plastic into energy or feedstock for fuels and chemicals. There are several methods to chemical recycling including pyrolysis and gasification [76,77]. However, the most commonly adopted method for plastic recycling is mechanical recycling; the outputs generally include clean and pellet-form recycled resins [75]. 

The recycling process starts with the collection of post-consumer and post-industrial plastic waste. Manufacturing and production firms often seek out collection services by recycling companies as means of disposal to meet environmental safety standards according to ISO standards [78]. Post-industrial plastic wastes are generally recycled more efficiently, as the plastic wastes are collected from each respective company, the source of the plastic wastes is typically the outcome of the processing of specific plastics, which do not require additional sorting. Kerbside collection is a form of post-consumer plastic waste collection method, plastic recycling firms often obtain licenses from local councils to set up collection plans for local households to collect general and recyclable household wastes [79]. Recycling companies also obtain plastic wastes from council regulated municipal waste collection drop-off centres that allows consumers to dispose of personal household plastic wastes as an alternative to kerbside collection. In some countries, post-consumer plastic wastes are sorted according to respective identification categories prior to kerbside collection. Most of the plastics being recycled and reused around the world are predominantly coming from post-industrial plastic streams rather than post-consumer. 

After being collected, the waste plastic is shredded. Large pieces of plastics are commonly shredded into smaller chunks and flakes before they are washed or decontaminated [80]. The process of shredding involves a series of rotating blades driven by an electric motor with specific grids for size gradation. Materials are fed into the shredder to produce coarse irregularly shaped plastic flakes [75,76]. As waste plastics come in various forms, predominantly categorised as rigid and flexible plastics, high-end industrial shredders can be designated for specific plastics [75]. However, flexible plastics require specific shredders, due to its soft and film-like form. Film plastics tear is more likely to carry contaminants within pockets of films [81]. 

The process following in the plastic recycling chain requires washing and decontamination. After being decontaminated, plastics are sorted into different categories. Traditionally, for large scale manufacturers, a method of separating polyolefins from common waste is to monitor density differences. Common polyolefins include plastics such as LDPE, LLDPE, HDPE, and PP that have lower density than water [80]. Therefore, polyolefins can be separated in a large tank of water by submerging waste and collecting whatever matter that floats on top. Plastics such as PVC are separated through X-ray fluorescent (XRF), where identifications of chlorine can be traced within the plastic [80]. Optical sorters utilise a series of Near Infra-Red (NIR) cameras to provide Hyper Spectral Imaging Technology. Upon contact with the plastics, the NIR wavelengths emit specific vibrations at a molecular level to indicate certain chemical compositions [75,82]. However, optical sorters can only differentiate certain types of plastics including PET, HDPE, PP, and PE. The excess is normally sorted to a ‘mixed’ category. Another method of sorting plastic involves the transfer of electrons from one particle to another. The electrostatic sorting method employs an electrical charge on to in-fed plastics on a conveyor belt specific to the materials [83,84]. The plastics take on a positive or negative charge reaching a high-tension field, they are then electrostatically separated into pure sorted fractions according to the different charges on each individual plastic particle [83]. 

Once separated, waste plastics are extruded. Extrusion is employed to homogenise and repurpose reclaimed plastics into convenient materials to work with. The process involves plastic forced along a tubular pipe and shaped through a die mould with an Archimedes screw. The plastics are input through a feeder and require to be in forms of flake, powder, or pellet. The Archimedes screw may vary in different measurements of diameter depending on the required output size. Similarly, the shape of the die mould can be designed differently and interchanged depending on the outcome requirement of the product. Heating coils are installed on the outer surface of the tubular pipe to ensure plastics are heated to optimum temperature to be shaped accordingly. The extrusion procedure precedes the quenching process where the plastics are cooled before pelletisation. 

The rate of cooling ultimately defines the structural properties of the pelletized plastics. Despite rapid cooling or ‘quenching’, process that cools and hardens the plastics rapidly, the process does not allow the modification of molecular orientation as there would not be adequate time for the chains to have free motion and form crystalline zones [85]. Water quenching and gas quenching are the two main methods employed in this procedure. Water quench involves the plastics to be inserted into a cold-water bath while gas quenching rapidly cools the plastics without oxidation to obtain a higher quality product [86]. Gas quenching is more expensive in comparison to traditional water quenching [87]. On the other hand, if the plastics are cooled at a slow rate, crystallization begins to occur, enabling the molecular orientation of the plastics to develop a more structured and defined form [88]. 

The final recycling step is the pelletizing which involves hardened plastics fed through an in-feed at a constant line speed, cut between a rotor and a bed knife into rough cylindrical pellets [89]. The size of the pellets will be dependent on the speed of the rotating blades. However, the shape of the pellets will be dependent on the shape of the extruder [90]. Plastic pellets can be subjected to post-treatment processes such as additional drying if the plastics have undergone a water quench, additional cooling if the plastics have undergone a slow cooling process, packaging, and storage. 

In combination with the ever-growing asphalt industry, road pavement technicians and scientists are adopting new techniques and methods to improve practices of construction and maintenance with a purpose to minimize damages on the current environment [91,92]. Evidently, the research of utilizing recycled plastics in the asphalt industry has significantly increased in the past decade. Research studies [10,16,61,93,94] in relation to using plastics on roads have found noticeable improvements in the road’s physical and mechanical characteristics namely, tensile strength, water resistance, durability, and overall life span, to name a few. 

Khurshid et al. [95] investigated the effect of addition of recycled LDPE and HDPE for bitumen modification in a physical analysis experiment. The mixing process concluded that HDPE was insoluble in bitumen even at 0.5% by weight, yielding a non-homogenous mix with prominent solids, while LDPE had a weak solubility in bitumen at mixing temperatures. As a result, HDPE was eliminated, leaving LDPE the only form of polymer utilised in the experiments. The results obtained shows an increase in penetration value with the addition of LDPE enhancing the overall stiffness. LDPE modified bitumen also exhibited a decrease in 16% in penetration value at 2% polymer content in comparison to conventional bitumen. The overall results concluded that the addition of LDPE increased softening point, flash point, and fire point, allowing a greater resistance against high temperatures. Nouali et al. [96] used LDPE from shopping bags to enhance binder characteristics. The results exhibited 15% improvement in softening point and increments in penetration index value while showing a reduction in temperature susceptibility. However, due to the poor compatibility of the bitumen phase and waste plastics, the storage stability was poor at high temperatures. This study also conducted research using the recycled LDPE-modified bitumen in comparison to conventional bitumen in a regular asphalt mix. The results showed that the recycled LDPE-modified bitumen exhibited an increase in water resistance, stiffness modulus and complex modulus by 13%, 20%, and 11%, respectively. Overall, the recycled-LDPE modified bitumen was proved to provide suitable workability and compaction ability for bitumen applications. Another study utilised recycled PE, PP, and PS for polymer modification. It was found that PE aggregate samples achieved the highest resistance against plastic deformation among the three selected polymers. The stiffness of the bitumen mixture was increased by 60% with the addition of recycled PE [97]. The following section discusses in detail about different type of plastomeric polymers (either virgin or recycled) from these categories, which have been widely utilized as a bitumen modifier in past studies.

## 5. Virgin and Waste (Recycled) Plastomers to Improve Bitumen Performance 

Plastomers are commonly used as bitumen modifier due to their lower cost—compared to elastomers—as well as their improved stiffness, consequently resisting to permanent deformation at high temperature; namely, rutting. One of the drawbacks in the use of plastomeric modification is the phase separation due to low compatibility of polymers with the bitumen, which results in two separate phases, i.e., asphaltene-rich phase and polymer-rich phase [98]. However, the storage stability is commonly a problem for chemically inert and non-polar plastics such as polyolefins, but when these plastics are co-mingled with polar substituents then the storage stability becomes a smaller issue. The mechanism of phase separation can be better understood by studying the polymer-bitumen interaction. During polymer-bitumen modification, the kinetically stable and thermodynamically unstable system is formed, where the polymer is swollen by the bitumen’s maltene fraction [99]. This thermodynamically unstable system persuades phase separation due to the influence of gravitational field, resulting in the settling of heavier asphaltene micelles at the bottom of blends during hot static storage [100]. Pérez-Lepe et al. [25] used high density polyethylene (HDPE) to modify the bitumen binder and found that the modification resulted in an enhanced high temperature performance and decreased storage stability, suggesting that such a type of modification is less effective for pavement applications. 

**Figure 3:**
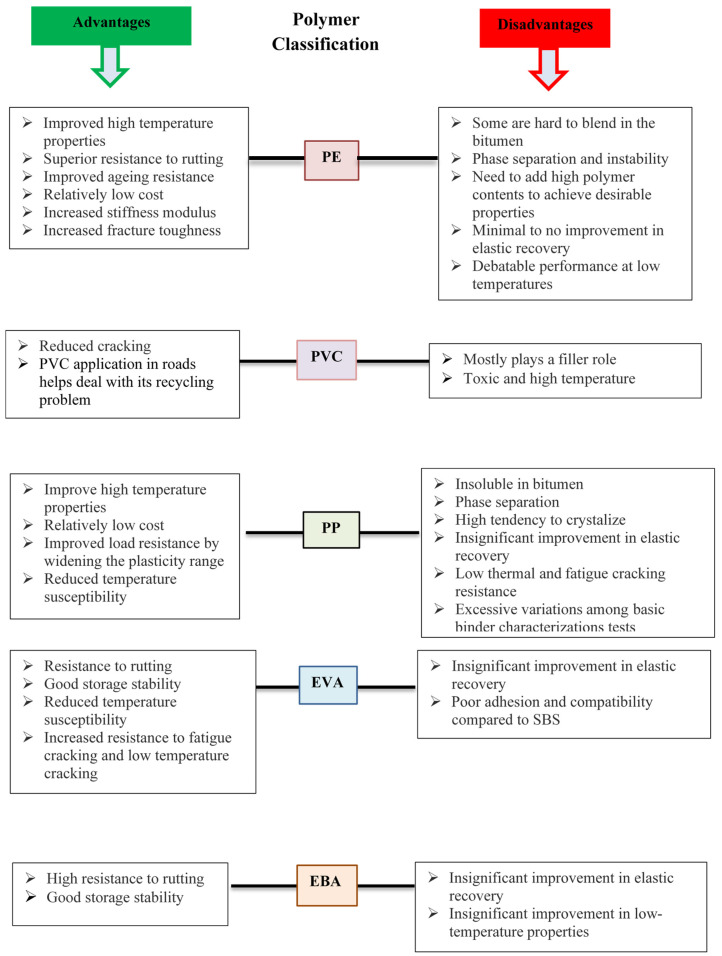
Advantages and disadvantages of different types of plastomers as modifiers for bitumen [105].

Phase separation is undesirable and limits the applications of these polymers for road pavement applications [65]; therefore, efforts have been put forward to avoid or minimize the phase separation and increase the compatibility between polymer and bitumen. The lower compatibility of plastomers is attributed to the nonpolar chains of the polymers. The compatibility can be improved by either removing or minimizing the non-polar groups, consequently adding polar groups by free radical polymerization with butyl acrylate or vinyl acetate, for instance, which tends to improve the compatibility of polymers with bitumen [101]. The addition of polar functional groups and substituents to the main nonpolar backbone by copolymerizing (e.g., EVA) or grafting (e.g., MA-g-PE) are known as a better solution to enhance the compatibility between plastomers and bitumen. On the other hand, it is reported that recycled plastics provide more polar groups as compared to virgin plastics because when plastic is being recycled it goes through a heating process, which causes ageing of the plastic itself. The polarity of the plastics increases with ageing [102], hence, recycled plastics offer better compatibility with bitumen. It is reported that phase separation of polymer-modified bitumen is influenced by storage conditions such as time and temperature, nature of bitumen binder, and polymer properties and concentration [100,103,104]. Polyethylene and polypropylene are the two most commonly used plastomers [9], other plastomers include ethylene-vinyl acetate, ethylene-butyl acrylate, poly(ethyl methacrylate), polystyrene and polyvinyl chloride [9]. The advantages and disadvantages of employing different plastomers for bitumen modification are shown in Figure 3. 

### 5.1. Polyethylene (PE) Modification of Bitumen 

Polyethylene, a long chain hydrocarbon derived from ethylene polymerisation, is a relatively cheap, thermodynamically unstable, and crystalline plastomer [105]. It exists in the form of high-density polyethylene (HDPE), medium-density polyethylene (MDPE), low-density polyethylene (LDPE), very low-density polyethylene (VLDPE), ultra-high molecular weight polyethylene (UHMWPE), and linear low-density polyethylene (L-LDPE) [12] depending on co-polymerization or branching, which varies its density and degree of crystallinity [9]. Metallocene-catalysed PE (m-PE), used to create mainstream PE such as LLDPE and HDPE, have also been used for bitumen modification. All different forms of PE can be evidenced in various objects [106] such as HDPE that can be found in several commercial containers (i.e., milk and shampoo), toys, pipes, and different houseware items; LDPE is found in containers and trays, reusable bags, and agricultural films, whereas L-LDPE can be found in objects such as geomembranes and food packaging films [9]. The basic characteristics of different forms of polyethylene are listed in Table 2. The density ranges between 943 and 961 kg/m^3^ for HDPE, 926–948 kg/m^3^ for MDPE, 890–953 kg/m^3^ for LDPE and approx. 910–940 kg/m^3^ for L-LDPE. The softening point ranges between 95 and 127 °C while the melting point of HDPE, MDPE, LDPE and L-LDPE ranges between 129–149 °C, 126–129 °C, 108–120 °C, and 124–128 °C, respectively (Table 2). As the melting temperature is commonly lower than the production temperature used to manufacture hot bitumen mixtures (i.e., 160–170 °C), therefore, these materials can potentially be incorporated in bitumen to obtain polyethylene-modified bitumen [12].

Table 3 and Table 4 list the mixing conditions and physical, chemical, rheological, and mechanical properties of virgin and waste plastomers modified binders, respectively. Polyethylene is commonly incorporated into bitumen at various percentages (0.1–6% for HDPE, 1–5% for MDPE, 2–10% for LDPE and 4–6% for L-LDPE) by weight of the binder, with mixing temperature ranging from 160 to 185 °C for HDPE, 165 to 170 °C for MDPE, 165 to 185 °C for LDPE, and 150 to 170 °C for L-LDPE; the mixing time is 0.5–6 h for HDPE, 0.2–1.5 h for LDPE, 0.5–1 h for MDPE, and 0.5–2 h for L-LDPE. The mixing speed falls within the range of 2500–4000 rpm for HDPE, 3000–4000 rpm for MDPE, 3000–5000 rpm for LDPE, and 4000 rpm for L-LDPE. The size of polymer added to the binder is also considered as an important parameter for homogenous mixing and the reported particle size for polyethylene-modified bitumen is 0.1–5 mm, as shown in Table 3. Studies suggest washing, drying, and extruding the waste polyethylene before trimming or grinding if it comes directly from post-consumer streams, or it can be directly trimmed or grinded to different sizes if supplied clean [66,146,147,148]. Post-industrial polyethylene is usually cleaner and of more consistent quality than post-consumer polyethylene. 

The addition of polyethylene affects the properties of modified bitumen blends as shown in Table 3. Polyethylene-based polymers have the potential to improve the in-service properties of asphalt mixtures such as resistance to high temperature rutting [104], high temperature behaviour, fatigue life, flexural stiffness, thermo-mechanical resistance, water resistance, adhesion, and elasticity [149,150,151,152,153,154,155,156,157,158]. The blending of polyethylene in bitumen tends to improve the glass transition peak of modified blends [159] and crystalizes from blends when it is cooled down because crystallites may crosslink extended polymer chains and form a gel network, which improves the high temperature stiffness until the crystals melt [31]. It is reported that polyethylene is immiscible with bitumen due to bitumen’s polar and aromatic nature and has less interaction with bitumen due to its tendency towards crystallization [105]. Although polyethylene-based polymers are insoluble in bitumen binders, they are still capable of flowing and spreading through the binder matrix (i.e., mechanical blending rather than chemical blending), hence improving the properties of the modified blend [160,161]. Different methods and techniques can be implemented to overcome the issues of less miscibility and compatibility of polyethylene-based polymers with bitumen; grafting and chlorination, for instance, are commonly used to disperse the polymer particles into the bitumen [162,163,164,165]. 

**Table 3:** Mixing conditions and physical, chemical, rheological, and mechanical properties of virgin plastomers-modified bitumen. Note: the symbol “-” identifies that no literature studies on bitumen provided quantitative indications of that specific property.

Mixing Conditions	LDPE	HDPE	LLDPE	PP	EVA	EBA	References
Polymer/binder percentage (%)	3–6	0.5–6	0.5–6	0.5–5	1–9	2–9	[35,36,123,140,166,167,168]
Mixing temperature (°C)	170	160–170	160–170	160–170	165–180	170–180	[34,36,118,123,140,166,167,168,169]
Mixing time (h)	1–2.5	1–2.5	1.2−2.5	1	2–7	2–6	[34,36,118,123,140,166,167]
Mixing speed (rpm)	4000	4000	4000	120	1000–3000	1000–1200	[34,35,36,118,140,166,167,169]
Physical Properties							
Softening point (°C) @ ASTM D-36	57–68.5	51–79	50–67	53–76	54–62	27–72	[34,36,125,140,168,169]
Penetration (dmm) @ ASTM D-5	23.5–40.8	21–36	13–41	15–35	47–53	46–75	[34,36,125,140,168,169,170]
Penetration index	0.44–1.17	−2–1.5	−2–1.5	1.96–2.28	0.49–1.24	0.07–2.92	[3,115,116,171]
Viscosity (cP) at 135–165 °C @ ASTM D4402	200–700	270–578	380	590–687.5	980	940	[8,34,44,115,170,172]
Ductility (cm) at 25 °C @ ASTM D-113	91–148.5	79–133	40.25–73.5	>100	5–22	10–40	[7,36,45,125,168,169,173,174]
Specific gravity @ ASTM D-70–76	1.014–1.042	0.935–1.01	-	1.015	1.015–1.032	-	[36,169,174,175]
Flash point (°C) @ ASTM D 92–02	200–240	215–257	-	199–292	260	-	[36,175,176,177]
Storage stability(softening point top–bottom)	0.8–2.5	0.96–1.1	3	-	1–1.9	0–3	[34,170,171,172,178,179]
Stability Index	48.1	5.42	8.43	-	-	-	[167]
Rheological Properties							
G*/Sinδ (kPa)	0.756–5.911	9–12.3	1.12–15.20	3.7–32.2	0.8–1.7	-	[163,170,171,180,181,182,183]
G’ (kPa)	-	-	0.31–29.90	0.38–7.04	-	0.62–4.94	[180,181,182,184]
G” (kPa)	-	-	6.4–37.5	3.7–30.6	-	0.71–7.57	
G* (kPa) at 10 rad/s	3.97–10.75	7.15–23.08	6.5–38.9	3.72–31.36	0.3115–170.790	62.3–75.0	
δ (°)	-	42.9–83.9	71.5–88.1	77.01–84.05	80–87	6.25–64.2	[180,181,182,183]
SARA Analysis (ASTM D-2006)							
Asphaltene (%)	-	-	17.6–18.8	11.1–13.6	11.7–14.7	-	[180,181,185]
Aromatics (%)	-	-	34.4–41.9	31.8–39.6	32.5–38.8	-	
Resins (%)	-	-	21.0–27.3	41.5–46.1	40.3–44.2	-	
Saturates (%)	-	-	17.3–19.7	6.8–8.1	8.6–9.2	-	

**Table 4:** Mixing conditions and physical, chemical, rheological, and mechanical properties of waste plastomers-modified bitumen. Note: the symbol “-” identifies that no literature studies on bitumen provided quantitative indications of that specific property.

Mixing Conditions	LDPE	HDPE	LLDPE	PP	EVA	EBA	References
Polymer/binder percentage (%)	2–10	3–6	2–5	3–6	1–3	N/A	[35,57,115,117,175,186,187,188]
Mixing temperature (°C)	160–170	185	180	165–170	180	N/A	[8,15,35,57,115,117,186,187,188]
Mixing time (h)	1–2	1.5	1.5	2	6	N/A	[8,35,57,117,175,186,187,189]
Mixing speed (rpm)	3000–5000	4000	3750	500	1800–4000	N/A	[8,35,57,115,175,186,187,189]
Physical Properties							
Softening point (°C) @ ASTM D-36	44–68.5	43.7–60.5	58–70	52.05–64	26	N/A	[8,9,115,175,176,186,189,190]
Penetration (dmm) @ ASTM D-5	41–74	46–68	47–56	27–68	37	N/A	
Penetration index	0.08–0.43	−1.7–0.6	−1.13–5.81	−0.8–2.28	0.11–2.38	N/A	[95,115,175,191,192,193]
Viscosity (cP) at 135–165 °C @ ASTM D4402	200–700	600	480	590–687.5	420	N/A	[8,115,189,190,194]
Ductility (cm) at 25 °C @ ASTM D-113	58–69	48–68	22–61	52–66	-	N/A	[176,189]
Flash point (°C)	200–240	215–257	-	199–292	-	N/A	[176]
Fire point (°C)	-	-	-	345	-	N/A	[175]
Storage stability @ ASTM D-7173(softening point top to bottom)	2.8–4.7	41.8	3.1–4.9	-	0.2–5.2	N/A	[8,189,191,195]
Rheological Properties							
G*/Sinδ (kPa)	0.09–12	2.26	1.12–15.20	2–47	4.08	N/A	[8,180,190,196,197]
G* (kPa) at 1 rad/s	1.23–11.7	-	6.5–38.9	-	-	N/A	
δ (°)	70.23–88.12	18	71.5–88.1	-	19	N/A	[180,190]
SARA Analysis							
Asphaltene (%)	19.0	15.4	-	-	11.7	N/A	[198,199]
Aromatics (%)	24.0	24.6	-	-	38.8	N/A	
Resins (%)	37.8	34.9	-	-	40.3	N/A	
Saturates (%)	19.2	25.1	-	-	9.2	N/A	

N/A: No data is available on polymer modification of recycled EBA.

**Figure 4:**
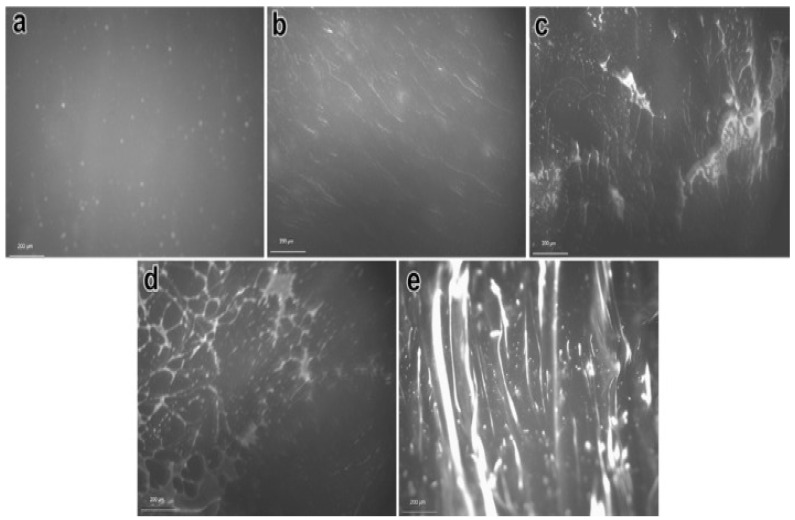
Fluorescent images of PE modified images at different concentrations of polyethylene (**a**) 2%, (**b**) 4%, (**c**) 6%, (**d**) 8%, and (**e**) 10% [204]. Reused with permission from Elsevier.

The blending conditions and composition of both polymer and binder have a significant effect on properties and performance of plastomers-modified bitumen blends. For instance, the melt flow index, an indirect indicator of molecular weight where higher MFI corresponds to lower molecular weight, is a polymer characteristic that portrays the architecture of the molecular structure of polymers [200] and affects the physical and rheological characteristics of plastomers-modified bitumen. It is suggested that a lower melt flow index resulted in a higher softening point and complex viscosity, lower penetration values, and that a higher mixing temperature increases the melt-flow index, resulting in increased performance of the polyethylene-modified bitumen. On the other hand, low mixing temperature causes incompatibility and dispersion instability and decreases the melt-flow index, hence decreasing the overall performance of blend [201]. It is reported that the molecular weight and distribution of molecular weight of polyethylene or polyethylene-based modifiers are important factors affecting the hot storage stability, low temperature properties, and phase separation of polymer-modified bitumen. Polyethylene-based modifiers with low molecular weight and wide distribution of molecular weight are considered suitable for bitumen modification purposes [202]. The concentration of the polymer also has significant effect on the performance of polyethylene-modified bitumen since high polyethylene concentrations (i.e., 5–15%) causes phase separation, hence 5% (wt%) has been suggested as a maximum limit for pavement applications [188]. Other studies suggested 4% and 6% as optimum weight percentage for polyethylene and polyethylene-based polymers [157,203]. The fluorescent images taken by a UV microscope of polyethylene-modified bitumen at different concentrations of polyethylene are shown in Figure 4a–e. Figure 4a illustrates the scattered spots of 2% polyethylene blended in bitumen; these scattered spots change to a filamentous structure as the concentration of polyethylene is increased from 2% to 4% (Figure 4b). A further increase in polyethylene content to 6% and 8% results in the formation of partly-developed or completed net-like structure (Figure 4c,d), whereas these lines thicken by further increasing the amount of polyethylene up to 10% [204].

The bitumen’s softening point increases whereas penetration decreases by increasing the concentration of polyethylene-based modifiers. As shown in Table 3, the reported range of softening point values are 44–68.5 °C, 50–67 °C and 51–79 °C for LDPE, L-LDPE, and HDPE-modified binders while the values of penetration for LDPE, L-LDPE, and HDPE are 23.5–79.1 (0.1 mm), 13–64.7 (0.1 mm), and 21–36 (0.1 mm), respectively. On the other hand, the standard softening point and penetration values of neat bitumen are 42–65 °C and 59.1–98 (0.1 mm), depending on the binder source and type (Table 1). The increase in softening point and decrease in penetration after addition of polyethylene-based modifiers shows greater consistency of the blends, possibly indicating higher resistance to permanent high-temperature deformations. A very common content used for polyethylene-based modifiers is 3%, which shows 7% to 42% reduction in penetration, whereas the penetration value is reduced by 22–63% for 5% polyethylene-based modifiers [12]. In relation to the softening point, an increase of 2–50% at 3% polyethylene content and 14–91.5% at 5% polyethylene loading was observed [171].

Viscosity is another important characteristic of bitumen and although a greater viscosity can provide improved performance at high temperature (i.e., resistance to flow), it can also hinder the workability and ease of construction of the asphalt mix. It was reported that the viscosity of polyethylene-modified bitumen is higher than that of conventional unmodified bitumen. In addition, viscosity increases by increasing the concentration of polyethylene-based modifiers due to the greater polymer-dominant phase in high-content polyethylene-modified bitumen [194,205]. On the contrary, ductility is decreased by incorporating polyethylene-based modifiers, hence indicating a possible brittle behaviour for polyethylene-modified binders at low temperature [125,174,205]. It was observed that the addition of polyethylene at 5% content resulted in decreasing the ductility up to 97% at 15 °C [206] and up to 35% at 25 °C [174,207]. The rheological characteristics of the polyethylene-modified bitumen suggest that a high concentration of modifier results in greater increments of the complex shear modulus (G*) and reduction of phase angle (δ) values, indicating a shift towards more elastic responses and increased stiffness [12]. Nizamuddin et al. [192] performed MSCR analysis of LLDPE modified bitumen and found that the % recovery was significantly increased whereas Jnr value was decreased by addition of LLDPE. However, the effect was more obvious at low percentages of LLDPE (3% and 6%) as compared to higher percentages of LLDPE. Beena and Bindu [41] added trans-polyoctenamer in LDPE and analysed both % recovery and Jnr value of modified samples; the study found that Jnr value was increasing and % recovery decreasing at lower percentage of additives although the opposite was observed at greater content of trans-polyoctenamer additive. Gama et al. [208] studied MSCR analysis of HDPE modified binder and found that % recovery of the neat binder was approx. 2.4% at 3.2 kPa and 15.7% at 0.1 kPa; these values were increased up to 91.5% at 3.2 kPa and 95.8% at 0.1 kPa, respectively, by adding HDPE, hence confirming the improvement of modified binder to maintain good elastic properties at high traffic levels and temperature. According to some studies, the storage stability of polyethylene modified bitumen is weak as it separates into two distinct phases (polymer-rich phase and bitumen-rich phase) during storage [160,163], even after short periods of time. However, other studies have found that polyethylene-modified bitumen can be considered stable at low to intermediate percentages of polyethylene, such as 5% of LLDPE [66] and 3% of HDPE or LDPE [194], by weight of bitumen. 

LLDPE is generally found to be more effective for improving the binder properties and low temperature performance as compared to LDPE and HDPE [118,160,209,210]. HDPE-modified bitumen commonly results in superior strength than LDPE, although the HDPE modification is prone to phase separation problems [25] due to the lower dispersion of HDPE into the bitumen phase compared to LDPE [211]. Fracture toughness of LDPE-modified bitumen was found to be higher than that of unmodified mixes at low temperature [212], which indicates an enhanced resistance to low temperature fracture [105]. Moreover, Marshall stability, fatigue life, resilient modulus, and moisture susceptibility were all improved by adding 2.5% of LDPE.

Metallocene catalysed polyethylene (m-PE) has been proposed as bitumen modifier because of its low cost, high dispersion characteristics, mechanical properties, physical properties, and improved storage stability [213]. It is suggested that the metallocene catalysis controls the molar mass distribution and molecular structure providing polymers with uniform distribution of short chains and narrow molar mass distribution [213]. This results in reducing the melt elasticity and tuning of bulk properties such as crystallinity and viscosity, consequently increasing the dispersion [214]. Spadaro et al. [214] used two different grades of metallocene catalysed LLDPE for blending with bitumen in order to validate the possible potential use of these polymers as bitumen modifiers. It was observed that the addition of metallocene-catalysed LLDPE enhanced both thermal and mechanical properties of modified bitumen. The glass transition temperature was significantly decreased by increasing the polymer concentration, suggesting advantages related to low temperature flexibility. The modified bitumen blends showed relatively low dynamic shear viscosity at higher concentration of polymers. Another study [213] investigated the rheological and thermal characteristics of metallocene-catalysed LLDPE blended with metallocene-catalysed HDPE. The results of zero shear viscosity, relaxation time and frequency show a linear variation throughout the weight fraction range, suggesting the miscibility of metallocene-catalysed LLDPE/metallocene-catalysed HDPE blends. A research study conducted by González et al. [210] studied the storage stability and viscoelastic characteristic of metallocene-catalysed LLDPE/bitumen blends. The metallocene-catalysed LLDPE/bitumen blends were prepared by loading 1–3% metallocene-catalysed LLDPE into bitumen at 180 °C, 1800 rpm, and 6 h. it was found that metallocene-catalysed LLDPE provided better storage stability by avoiding phase separation at high temperature of storage.

### 5.2. Polyethylene Terephthalate (PET) Modification of Bitumen

PET is most found in water bottles and food packaging and is mostly intended for single use applications although major companies are now reusing up to 100% waste PET to produce new plastic bottles and other products. PET was found as a suitable candidate for its application in asphalt pavements due to its strong chemical resistance to organic materials and water, its high strength-to-weight ratio that makes it a suitable plastic in the asphalt industry to reduce environmental pollution and improve bitumen material characteristics. Unfortunately, PET and recycled PET are very expensive polymers—especially when classified as ‘food grade’—and their uses are predominantly made for containers used in the food industry. The very high melting temperature of PET (i.e., approx. 260 °C) also limits its application as bitumen modifier. However, different studies have reported that the addition of PET as a polymer modifier for bitumen enhances various properties of the mix. Recently a review article was published on application PET for pavement performance [215]. El-Naga and Ragab [216] used PET for bitumen modification and studied its effect on the overall properties of PET-modified bitumen and PET-modified asphalt mixes. The PET modification tends to decrease penetration and increase softening point, ductility [217] and viscosity [218]. Results also showed higher Marshall stiffness modulus, indirect tensile strength and rutting stiffness in comparison to conventional modifiers used in asphalt mixtures [219]. The value of air voids and voids in mineral aggregates increased with the content of PET in the modified asphalt mixture [220]. In addition, the rheological study conducted by Bary et al., found that 4% PET enhanced the complex shear modulus and decreased the phase angle significantly [221]. However, since PET does not melt during the bitumen modification process (i.e., can only act as a filler), its relative size should be carefully considered during DSR tests, especially when a 1 mm gap is used. The PET modification of bitumen improved the G*/sinδ value, indicating better performance against rutting [222]. Leng et al. [223] studied MSCR characteristics (%recovery and Jnr) of PET modified bitumen binder at 0.1 kPa and 3.2 kPa. It was observed that the %recovery of modified binder was significantly higher and Jnr value of modified binder was significantly lower than those of pure binder. In addition, the %recovery increased and Jnr decreased by increasing the polymer content. Al-Jumaili [224] utilised PET (from 2.36 mm to 4.75 mm), crumb rubber (same size of PET and added through the dry process) and waste engine oil for bitumen modification. Results showed that 9% crumb rubber content achieved the greatest tensile strength and highest resistance to water damage. The increment of tensile strength values peaked when a greater percentage of PET was added into the bitumen. Overall, the Marshall stability was improved at 9% crumb rubber, 12% PET, and 5% waste engine oil. The study however did not investigate the possible lack of adhesion between the PET particles and bitumen. Another study [225] compared the fatigue properties of SBS and PET modified bitumen and found that both SBS and PET mixes improved the fatigue response but SBS blends showed better fatigue behaviour than PET blends. According to Cong et al. [226] fatigue behaviour is proportional to the surface energy, as the greater the surface energy, the higher the fatigue life will be. It was also found that the surface energy of SBS modified binder was higher than that of the neat bitumen, hence, SBS modification resulted in improved fatigue life. Arguably, in the same study, PET-modified blend showed higher fatigue behaviour than SBS modified bitumen at 6% PET loading and low strain level. Similarly, it is suggested that PET modification of bitumen has an effect on low temperature cracking, i.e., PET-modified bitumen blends are more susceptible to thermal cracking [227]. The mixing process of PET with bitumen also plays an important role on the overall performance—as discussed earlier there are two approaches for mixing polymers with bitumen, dry process, and wet process. However, the wet process is not considered feasible for PET due to the very high melting temperature of PET, which makes it difficult to achieve homogenous blends [228]; however, there are studies that reported on PET modification of bitumen by wet process. Moghaddam et al. [219] studied the wet mixing of PET and bitumen and found that permanent deformations of PET modified bitumen were improved significantly and a higher PET amount showed better resistance against permanent deformation. Choudhary et al. [228] reported of a dry process and modified dry process (initially heated aggregates were mixed and coated with PET and then bitumen was added and blended) for PET modification of bitumen and found that the modified dry process showed better performance and had high resistance towards moisture-induced damage. Generally, PET-modified bitumen from either of the processes showed greater resistance against deformation with high stability, low flow, and high Marshall quotient.

### 5.3. Polyvinyl Chloride (PVC) Modification of Bitumen

PVC shares about 10.1% of the European plastic production. It is commonly used for the manufacturing of profiles, cable insulation, garden hoses, and window frames [229]. PVC is dubbed as poison plastic because it contains various toxins. Incineration or firing of PVC-based products causes dioxins to be produced [230]. It is reported that PVC emits hydrochloric acid (HCl) in large quantity when it is heated at high temperature. It is suggested that PVC can emit HCl even though it is not actually ignited in a fire. HCl produced by heating of PVC can cause widespread damage to the instruments and environment due to its toxic nature [231]. To deal with the high chlorine content of PVC, it is suggested to partially remove chlorine from PVC through chemical treatment. It is reported that chlorine displacement from the surface of PVC is possible by nucleophilic substitution of ligands such as amines and hydroxy [232]. Considering the 30-year life span of PVCs, there is no absolute safe way of dealing with this hazardous polymer. Additionally, the collection, transportation, and disposal methods are still not conducted properly, especially in developing countries. Uncontrolled dumping of such wastes leads towards serious environmental concerns including ground water pollution [230]. Some studies tested PVC as polymer modification of bitumen although they could not get successful results due to the polymer high melting point (i.e., approx. 298 °C) [194,197,233]. Some other studies used PVC from different origins including window frames, cables, pipes—or not mentioning the origin of PVC—for bitumen modification with limited outcomes [230,234,235,236,237]. The mixing conditions for PVC modification of bitumen included 140–190 °C mixing temperature, 20 min to 3 h mixing time, 0.075–2 mm particle size, 1–20% concentration of PVC by weight of the bitumen, and 1300–3750 rpm mixing speed [12,230,234,235].

Although used as a filler to create PVC-based bituminous mastics, it was found that the addition of PVC to bitumen improved the conventional and rheological properties. The penetration value generally decreased whereas the softening point value was significantly increased after PVC addition to the base bitumen. Five percent PVC into the bitumen reduced the penetration value by 57% and increased the softening point by 26% [12]. Viscosity was increased up to 300% while ductility decreased after addition of 5% PVC in bitumen [236,238]. From the rheological perspective, it was noticed that PVC modification of bitumen resulted in improving the complex shear modulus and reducing the phase angle of the PVC-modified bitumen [230,235]. The increased complex shear modulus and the decreased phase angle helped enhance rutting resistance at high temperature, perhaps indicating greater durability of pavements [12]. There were only a few studies that used additional modifying agents with PVC to modify the base bitumen. For instance, Fang et al. [238] used 0.05%, 0.15%, and 0.25% organic montmorillonite as an agent to improve the properties of PVC-modified bitumen and it was found that the storage stability was improved, specifically at 5% PVC. Another study utilized a chemical modifier with PVC to improve the properties of modified bitumen and found that the dispersion of the polymer in bitumen was improved significantly [230]. No mention in the study was made about the potential fuming and emissions during high temperature heating of PVC.

### 5.4. Polypropylene (PP) Modification of Bitumen

Polypropylene (PP), currently accounting for 21% of the global plastic production [239] and 19.1% of the European plastic production [229], is commonly found in automotive parts, microwave-proof containers, food packaging, and pipes [229]. PP is a thermoplastic linear hydrocarbon with an intermediate crystalline level between that of HDPE and LDPE [105]. PP has been extensively used as a polymer modifier for bitumen in order to improve its properties. It is reported that PP-modified bitumen blends show increased resistance to rutting and fatigue life when included into the bitumen mixture; PP is also ascribed as to improve stability, Marshall properties, and indirect tensile strength [240,241,242,243]. The mixing conditions of PP with bitumen reported in literature are as follows; 165–180 °C mixing temperature, 1–2 h mixing time, 120–4000 rpm mixing speed and 1–6% concentration of PP by weight of the binder (Table 3). The most common dosage values of PP used for polymer modification of bitumen are 3% and 5% [12].

It was found that incorporation of PP into bitumen significantly varies the properties of PP modified bitumen. PP modification of the binder resulted in increasing the softening point while decreasing the penetration value. As mentioned earlier, 3–5% are the most commonly used PP concentrations; the penetration value was observed to drop between 18 and 30% for 3% PP and between 38 and 50% for 5% PP [12]. On the other hand, 4–30% and 11–43.5% increase in softening point was observed for 3% and 5% of PP, respectively [12]. Viscosity was increased and ductility was reduced after the addition of PP into bitumen, and the ductility was diminished by 20% at 5% PP content [244]. Such observations indicate that there are various factors including particle size, mixing duration, and interaction between the binder and modifier, which should be considered before utilizing such kinds of polymers for bitumen modification. In terms of rheological properties, PP modification of bitumen improved the rheological properties of PP-modified bitumen at high temperature and low frequency, resulting in an increased resistance against permanent deformation. The complex shear modulus was increased, the phase angle was decreased, and greater G*/sinδ values were achieved after PP modification of bitumen [12,245]. Different forms of PP, including aPP, iPP, and sPP, have been successfully employed for bitumen modification [30,246]. Nekhoroshev et al. [247] assessed the aPP modification of bitumen binder and found that adhesion properties were improved, however, aPP has low-crosslink density. Al-Haidri et al. [248] studied the effect of two different grades (atactic polypropylene, aPP, and isotactic polypropylene, iPP) of PP modifiers and found that both iPP and aPP resulted in increasing the resistance to stress, consequently reducing the number of distresses and increasing the in-service life of pavement. However, aPP at 2% concentration provided better results as compared to iPP. Schaur et al. [249] added four different PP polymers including two isotactic polymers with different molecular weights, one isotactic polymer with polar (anhydride) side groups, and one atactic polymer. They found that the long-chain iPP represented heterogeneously distributed polymer whereas the short-chain polymer was finely dispersed. The aPP has less impact on mechanical characteristics of PMB as compared to iPP, establishing a partial network of polymer-rich phase in PMB whereas maPP improved mechanical properties significantly. Awad and Awad and Al-Adday [250] investigated the utilization of PP as a bitumen modifier and found that PP resulted in greater stability as compared to conventional bitumen in the absence of PP. Various studies reported about storage stability and suggested that the use of PP leads towards phase separation [194,245]. However, to limit this shortcoming, Giavarini et al. [101] suggested adding 3% of polyphosphoric acid (PPA) by weight of bitumen to PP and found acceptable storage stability values. Other modification methods include the incorporation of maleic anhydride to PP and pyrolysis products of PP before using it for binders modification [12]. Although there is a general lack of studies addressing the fatigue performance of PP-modified asphalt mixes, PP has been proved to be a reasonably good polymer—both in its virgin and waste form—to modify bitumen.

### 5.5. Polystyrene (PS) Modification of Bitumen

The global market for PS has been on the climb, increasing its compound annual growth rate (CAGR) by more than 5.5% since 2010 [251]. In comparison, the demand of PS from 2000 to 2010 only grew at 1.4% rate in CAGR. In 2019, the PS global market was recorded at $42.7 billion and with its current rate of growth, the global market for PS is expected to increase to 9.8% in CAGR by 2023, reaching over $62 billion. PS is extensively utilized for manufacturing of disposable containers and packaging materials. PS is produced by polymerisation of the monomer styrene and is non-polar as it only contains carbon-hydrogen bonds. It is conveniently recycled multiple times with minimal reduction of its initial properties [252].

Recently, it has been utilized as a modifier for bitumen to form PS-modified bitumen, providing superior conventional physical properties [253]. Fang et al. [254] used expanded polystyrene (EPS) from the packaging industry for bitumen modification and found that the addition of EPS improved viscoelasticity and rutting resistance. A research work was conducted where 5%, 10%, and 15% of PS was added to bitumen and the authors found that the amount of PS has a direct effect on properties of modified bitumen by increasing the softening point, fire point, and flash point and decreasing ductility and penetration [253]. An increase in the softening point at the increasing content of PS shows an improvement in high-temperature stability with the addition of EPS [255]. The effect of extruded PS waste on viscosity of modified bitumen was investigated by Abinaya et al. [256] and it was found that the viscosity increased by increasing the content of PS. Johnson et al. [257] prepared PS/bitumen blends at 1%, 2%, and 3% loading concentration of PS at 180 °C, 600 rpm for 1 h. It was found that the softening point of modified bitumen was increased by 29% for 80/100 grade bitumen and 35% for 60/70 grade bitumen by the addition of PS. The penetration value was decreased up to 20% due to addition of PS. It was concluded that PS increased binder resistance to temperature change as well as the flow resistance, thus indicating enhanced rutting resistance of PS-modified bitumen. It was also observed that the addition of PS in bitumen could potentially provide high resistance against rutting and fatigue as compared to virgin bitumen.

Although PS improves various properties of modified bitumen, it also has some drawbacks such as low mixing compatibility, storage stability problems, and poor elastic and low-temperature properties [258]. Another study proposed the incorporation of nano-clay at 2%, 5%, and 10% weight of PS and observed that the percentage of nano-clay increased the thermal stability and rheological properties [259]. Padhan et al. [258] studied the MSCR performance of PS-modified bitumen with the addition of trans-polyoctenamer at stress levels of 0.1 kPa and 3.2 kPa. It was found that at both stress levels, Jnr decreased continuously whereas %recovery increased due to PS; the addition of trans-polyoctenamer seemed to suggest that it provides a better recovery of the PP-binder from deformation during loads. 

PS is one of the susceptible polymers when it is exposed to the sun (specifically, UV radiation); however, there are very limited studies on UV degradation of PS. UV degradation of PS leads to excessive embrittlement of the plastic, hence potentially leading to significant cracking pattern on the samples. The degradation, embrittlement and cracking of PS when exposed to the sun (UV) can considerably restrict its application as a bitumen modifier. Therefore, it is recommended to study UV ageing of PS modified binders.

### 5.6. Ethylene-Vinyl Acetate (EVA) Modification of Bitumen

EVA is a thermoplastic polymer formed by co-polymerization of vinyl acetate and ethylene [133], where the vinyl acetate content is controlled through a copolymerization process [260]. EVA has been used in the modification of bitumen for decades due to the enhancement of molecular bonding between VA and carbonyl linkages that affects the polarity between the two materials [169]. The basic characteristics of EVA reported in different literature studies are provided in Table 2. EVA is similar to LDPE in terms of rigidity and translucency; depending on the VA content EVA can be transparent like rubbers or plasticized PVC and can resemble elastomers for flexibility and softness. EVA is a form of plastomeric polymers used for modification of bitumen where the variation in vinyl acetate content provides interesting characteristics to EVA-modified bitumen. It is suggested that the higher content (i.e., greater than 20% VA) of vinyl acetate in EVA enhances the polarity of the polymer and the elasticity, storage stability, and flexibility of EVA-modified bitumen whereas lower content of vinyl acetate of EVA (i.e., 8–14% VA) increases the degree of crystallinity, leading to stiffer EVA-modified bitumen with enhanced high temperature performance [261]. EVA-based polymers form a rigid and tough bonding with bitumen during its modification to resist the deformation [33]. Unlike many plastomers discussed in the previous sections, EVA—also depending on the VA content—tends to improve the thermal cracking at low temperature and permanent deformation [35].

The mixing temperature for preparing EVA-modified bitumen is commonly 160–200 °C, as shown in Table 3. The lower limit of the temperature range is generally selected at approximately 160 °C due to the fact that the homogeneous mix of EVA in bitumen is difficult to obtain at lower temperature, even after 1 h of mixing time [169]. It is reported that the variation in mixing temperature does not seem to significantly affect penetration, ductility, specific gravity, and softening point of EVA-modified bitumen [169]. The effect of mixing duration for EVA-modified bitumen is listed in Table 3. The overall mixing time reported for mixing of EVA and bitumen is between 20 min to 6 h (Table 3). A study investigated the effect of mixing time on various physical properties of EVA-modified bitumen at 20, 25, and 30 min mixing time and observed that there is no significant change in the physical properties of the final bitumen [169]. Various concentrations of EVA have been used for bitumen blends. Concentrations of EVA at 1–10% are used in different studies (Table 3), where 5% by weight of the bitumen is found to be the most commonly used percentage of EVA. Panda and Mazumdar [169] studied the effect of different concentrations of EVA by varying its loading from 2.5% to 10% by weight of the base bitumen. It was observed that increasing the polymer concentration improves the softening point and lowers ductility and penetration. Another study found that 5% EVA caused an increase of 21.6–53% in softening point and decrease of 33–51% in penetration [12]. However, the increasing level of softening point varies by varying the concentration of the polymer. For instance, the softening point of EVA-modified bitumen as compared to base bitumen increases by 14 °C, 16 °C, and 18 °C at 4%, 5%, and 6% concentration of EVA, respectively [116]. An enhancement in the softening point (showing a stiffening effect of polymer modified bitumen) by addition of EVA polymer can favour the pavement durability in warmer climatic conditions. Similarly, penetration index and ductility values were improved by adding EVA at increasing concentration, which suggest that EVA-modified bitumen is more resistant to rutting and low temperature cracking. The results of storage stability of EVA-modified bitumen indicates that the temperature difference between the top and bottom portion of the cigar tube test (ASTM D7173) sample was less than 2.5 °C, confirming that EVA-modified bitumen is mostly stable at higher temperature [105]. Further, it was suggested that some sort of separation may occur during storage of EVA-modified binders, but EVA gets easily dispersed in bitumen and has relatively high compatibility with binders. EVA with low VA content is expected to provide high storage stability [201]. In order to further improve the storage stability of EVA-modified bitumen, some stabilizing agents were introduced with EVA, which helped enhancing the overall characteristics of binder. Among others, innovative stabilizing agents include maleic anhydride and nano clays. Addition of maleic anhydride helps decrease penetration, improves storage stability, and increases the ductility and softening point [116]. On the other side, the nano clays result in increasing the softening point and provides stability to binders up to 6% of EVA; the latter content was not stable in the absence of the nano-clay additive [262]. 

The viscosity of bituminous binders at in-service temperature of asphalt is generally expected to be high to avoid rutting (permanent deformation), whereas it should be very low at handling and manufacturing temperatures, resulting in an easy construction of the pavement. It was observed that the addition of EVA raises the viscosity value when EVA content is greater than 3% [35]. The ductility of bitumen was increased after addition of EVA; for instance, 5% EVA resulted in 20% increment in ductility, which contrasts with other polymers such as PP, PVC, and PE. The higher content (9%) of EVA polymer results in the rheological behaviour of modified bitumen showing better performance as a paving binder [29]. It was found that the phase angle decreases whereas the complex shear modulus increases by increasing the concentration of EVA. It is suggested that the testing temperature, concentration, and nature of the polymer have a significant effect on storage modulus and loss modulus of the polymer-modified bitumen [68]. Liang et al. [261] studied the MSCR of EVA-modified bitumen and found that the VA content in EVA offers higher %recovery whereas increasing temperature and stress load cause a sharp decline in %recovery, meaning that higher temperature associated with heavy loads degrade the recoverable ability of asphalt pavements. Similarly, the VA content of EVA has a significant effect on Jnr value of EVA-modified bitumen due to EVA crystallisation temperature.

**Figure 5:**
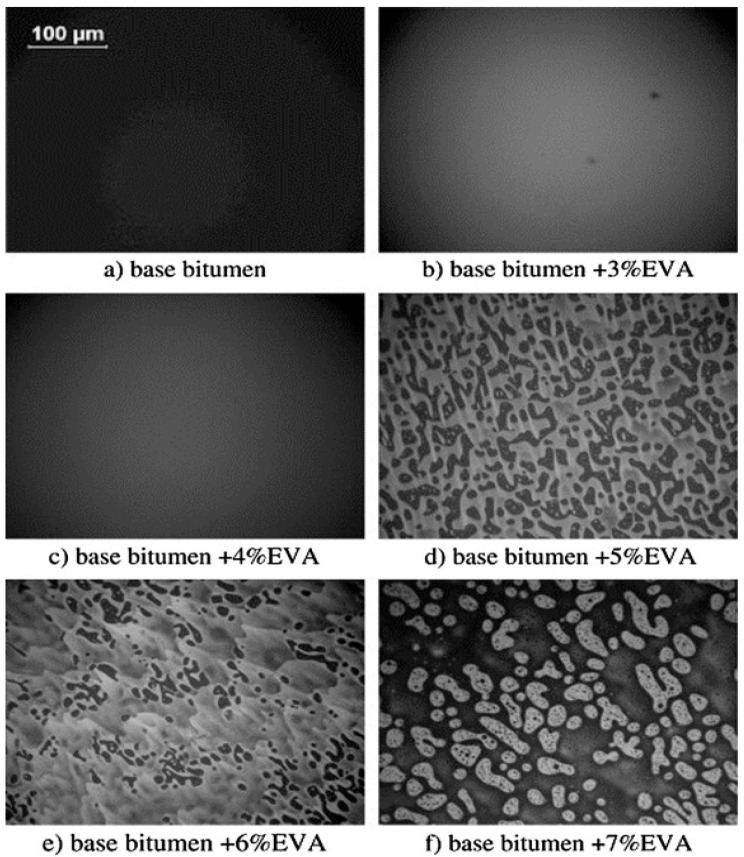
Fluorescent images of EVA-modified bitumen: (**a**) base bitumen, (**b**) 3% EVA, (**c**) 4% EVA, (**d**) 5% EVA, (**e**) 6% EVA, and (**f**) 7% EVA [33]. Reused with permission from Elsevier.

The thermogravimetric/differential thermogravimetric (TG/DTG) analysis of base bitumen and EVA-modified bitumen suggests that the TG decomposition stage for bitumen happens at 290–490 °C with 75% mass loss, hence decomposing the asphaltenes to form coke [116]. The DTG curve shows that the maximum weight loss took place at 450 °C. On the other hand, the TG curve for EVA modified bitumen highlights a decomposition peak at 307–492 °C and 462 °C for the DTG curve. The initial and maximum decomposition temperatures of EVA-modified bitumen are higher than that of base bitumen, suggesting that the addition of EVA resulted in increasing the thermal stability of bitumen [116]. The morphological images of EVA-modified bitumen are shown in Figure 5. It can be observed from Figure 5 that the EVA-modified bitumen showed fine dispersion of polymer, which is confirmed by the presence of the polymer network at 5–6% of EVA content [33].

## 6. Co-Mingled Plastomers for Bitumen Modification

Plastics recovered from the post-consumer stream contain many different types of plastomers. As a result, they contribute to most of the co-mingled recycled/waste plastomers in this case [263]. As discussed in the previous sections, each plastomer—whether chemically pure or recycled—has some specific drawbacks, hence, various studies tried to combine different polymers in order to improve the properties of modified bitumen. Various research studies have reported different combination of polymers (mainly elastomers and plastomers) for evaluating the performance of both individual polymers and combined polymers [206,264,265]. The combination of two polymers is carried out either by extruding the polymers before they are added into the bitumen [266,267] or simply by adding the single polymers in a defined proportion and then mixing them with the bitumen [206]. Most of the studies have focused on the combination of elastomers and plastomers such as tyre rubber and PE [268,269], tyre rubber and EVA [270], tyre rubber and PP [265], tyre rubber, EVA and PE [267], and styrene butadiene styrene and PE [271]. Brovelli et al. [266] used two plastomers (i.e., PE and EVA) in combination. However, the most common combinations investigated involve tyre rubber as an elastomer and any of the plastomers to enhance the characteristics of a waste (or recycled) polymer. The addition of rubber with PE-modified binders is suggested to be an effective way of improving both the low and high temperature properties of the blend while maximising the use of recycled products [105]. When combined together, PE helps to enhance the high temperature properties by stiffening the modified bitumen whereas the rubber tends to improve the low temperature properties [161]. It is reported that physical interaction occurs between rubber and PE during combined modification, which helps for homogeneous distribution and size refinement of polyethylene [105]. 

The mixing conditions for bitumen modification of comingled polymers vary depending upon the type of polymer used. Generally, the mixing temperature found in several studies dealing with co-mingled plastomeric polymers varies between 120 °C and 190 °C, mixing time ranges between 0.5 and 6 h, and 1200–5000 rpm mixing speed [2,265,267,272]. A study by Yuan et al. [273] reported the highest mixing speed of 25,000 rpm with 7–12% of PE, while another study employed a combination of PE and EVA at a mixing speed of 1200–5000 rpm for 6 h [267]. Generally, the penetration value is decreased at a combination of tyre rubber and PE/PP as compared to the base binder but the reduction in penetration value in presence of tyre rubber and plastomer is higher than that of only tyre rubber and lower than that of only plastomer [12]. On the other hand, the changes in softening point commonly exhibit a steeper upward trend with the combination of two polymers compared to the individual polymers. The addition of PE and tyre rubber decreases the ductility of modified bitumen up to a fixed amount of PE, while when the amount of PE is less than tyre rubber, the blend shows improved ductility [206,264]. Adding stabilizing agents—such as furfural extract oil [265], reactive dioctyl phthalate [265], polyphosphoric acid [208], sulphur [274] and maleic anhydride—to the combination of polymers before blending with bitumen generally increases the ductility of the modified bitumen. A study reports that ductility was increased by using maleic anhydride grafted LDPE with styrene butadiene styrene and HDPE before adding them into the bitumen [40].

In terms of rheological properties, it is reported that the addition of hybrid (or combined) polymers tends to enhance the rheological characteristics of modified bitumen by increasing the complex shear modulus and reducing the phase angle [206,275]. A combination of PE with other polymer increased the Superpave parameter G*/sinδ [208,273], hence indicating greater resistance to rutting. The combination using maleic anhydride grafted LDPE resulted in decreasing the phase angle and improving G*/sinδ [40]. The addition of a large amount of tyre rubber with plastomers enhances the mechanical characteristics, indicating betterments for low temperature flexibility and resistance to high temperature deformation [265,268]. Regarding storage stability values, mixing 2% styrene-butadiene-styrene to 6% PE (HDPE and LDPE) or mixing 5% styrene-butadiene-styrene, 3.5% PE and 3% furfural extract oil showed good storage stability values [233,275]. Further, the addition of maleic anhydride grafted LDPE to styrene-butadiene-styrene, LDPE and HDPE decreases the phase separation, consequently increasing the compatibility and storage stability [40]. Appiah et al. [276] conducted a physical and rheological study by utilising a combination of HDPE and PP for bitumen modification. The results exhibited an increase in the softening point, a decrease in penetration value while enhancing the overall dynamic and absolute viscosity of the binder in comparison to conventional bitumen. Although no distinct or new findings were observed in the spectroscopic analysis, the study showed a strong bond between polymer strands and the bitumen matrix. Another study investigated the effect of individual PP and HDPE as well as a combination of PP and HDPE at 1:1 ratio and were added to bitumen at 9% weight of bitumen. Marshall test results suggested that HDPE/PP modified bitumen showed 179% improvements in Marshall stability, whereas PP and HDPE increased its value to 156% and 152%, respectively [277]. 

Manju et al. [278] examined the effect of utilizing combined and recycled polymers including PVC and HDPE pipes, LDPE plastic bags and PET bottles as modifiers for bitumen. Results showed a 40% and 9% decrease in crushing and impact values, respectively, suggesting an increase in strength. The penetration and abrasion test witnessed lower values obtained in comparison to the control sample. The study concluded that the recycled polymer-modified bitumen has shown greater stability in comparison to conventional bitumen. According to Mahmuda et al. [279] hybrid combination of PET and PS showed some potential to enhance the rutting resistance of asphalt. Nkanga [280] studied the incorporation of PET and PS at 1:1 ratio for bitumen modification. A combination of PS and PET increased the specific gravity, softening point, flash, and fire points, while penetration and ductility were decreased. The study concluded that PET and PS improved the road’s durability, resistance to deformation, corrugation, and shoving. Another study employed LDPE, PS, and a blend of both polymers at 1:1 ratio and found that an increase in concentration of polymer from 5% to 15% improved the Marshall stability, flow, bulk density, strength, and fatigue life of the asphalt mix [280]. A study conducted by Chowdhury [281] utilised a combination of polyethylene and polyester; the outcomes showed that the optimum polymer content was 8% by weight of bitumen. The mixing process exhibited a glue and binding effect that improved the physical properties of the aggregates significantly. The stability and ITS (wet-dry, freeze-thaw) of the overall mix increased due to the plastic modification of the mix, specifically on the bond between aggregate and bitumen with the addition of plastic. Yan et al. prepared a blend of bitumen by combining two polymers (tyre rubber and PE) and observed that the modified binder showed uniform distribution between bitumen and polymeric materials [206]. A continuous bitumen phase with dispersed polymer particles by adding a single agent was observed, whereas with the addition of CR and PE under integrate modification, a nearly continuous polymer phase appeared and finally formed two continuous twisted phases [282]. 

Although comingling of two polymers enhances various properties of modified bitumen, there are some drawbacks which require further investigation—such as low temperature rheological behaviour. In fact, combined polymers could limit the bitumen’s ability to relax thermal stress [206]. In the case of crumb rubber being one participant polymer, complex chemical reactions take place during blending and thermal dissociation (or depolymerisation) of crumb rubber can occur [283]. It can be observed that the addition of combined polymers to bitumen has the potential of increasing phase separation due to less compatibility of these polymers in the bitumen. Hence, different additives have been utilized for polymer modification of bituminous binders in an attempt of enhancing the compatibility between bitumen and polymers. The following section discusses the different chemical compatibilizers used for polymer modification of bitumen. 

## 7. Enhancements of Plastomer-Modified Bitumen Due to Chemical Modifiers 

Many studies tried to address common plastomer drawbacks such as phase separation and thermal instability. Polymers and bitumen become less compatible due to differences in their structure, molecular weight, density, polarity, and viscosity. A difference in density may cause the creaming of polymer particles, which leads to phase separation. Together with the physical blending between bitumen and polymers, chemical modification is also suggested to overcome common drawbacks and improve the properties of modified bitumen. Chemical modification utilizes various chemicals as an additive to the bitumen, extenders of the binder, or modifier to the binder; this section focuses on chemical modifiers to stabilize plastic-modified bitumen. It is suggested that the polymer modifiers should be highly compatible to the binder, hence resulting in homogenous blends and minimizing the phase separation during transportation and storage [105]. Different modifiers play different roles in improving the properties of polymer-modified bitumen; for instance, maleic anhydride improves polarity and decreases crystallinity, which disrupts closely packed crystalline microstructure [99] resulting in increased compatibility and storage stability [100,163,171], organic montmorillonite supports chemical bonding between the bitumen and polymer and polyphosphoric acid enhances the rheological characteristics of the binders at high temperatures [194,284]. To date, various modifiers have been employed to obtain the required compatibility and reduce the phase separation during polymer modification of bitumen. Among the most common modifiers: reactive polymers, polyphosphoric acid, organometallic compounds, sulfonic acid, silanes, maleic anhydride, carboxylic anhydride, thiourea dioxide, sulphur, antioxidants, nanomaterials, clay minerals, plasticizers, and bio-oil [4,105,162,205,210,285,286,287,288,289]. Table 5 lists different the chemical modifiers used for plastomeric bitumen modification.

### 7.1. Chemically Functionalized Polymers 

The shortage of butadiene in 2009 hit the SBS market affecting the cost and supply of the most commonly used polymer in bitumen [290]. The high cost of SBS pushed companies to find an economic alternative to SBS, consequently, reactive polymer was recognised as a promising option to potentially substitute SBS [291]. The functionalization of polymers with new functional groups (i.e., acrylic acid and glycidyl methacrylate) leads to new polymer modifiers; i.e., reactive polymers which may crosslink or chemically bond with bitumen molecules through their functional groups [105,292]. In functionalization, different chemical functional groups are added to the polymer for improving the properties of polymer-modified binder, specifically tackling the compatibility of the polymer phase with the bitumen phase for minimizing phase separation. Some examples of such polymers are thermoplastic elastomers functionalized with maleic anhydride and ethylene-based copolymers with epoxy rings [67]. Reactive polymers are mostly based on glycidyl-methacrylate, ethylene, and ester groups—either ethyl, butyl or methyl acrylate, amorphous poly alpha olefins, and trans-polyoctenamer.

Reactive polymers can potentially be utilized as polymer modifiers [293], bitumen modifiers [294], and compatibilizers between polymer and bitumen [57] for improving the mechanical properties, temperature susceptibility, and storage stability of modified bituminous blends and bitumen mixtures [105]. These polymers have a unique characteristic of chemically linking to bitumen to develop a chemical bond with bitumen molecules [65,116]. Reactive polymers contain various functional groups which react by forming a three-dimensional (3D) bonding. This 3D bonding tends to improve the performance and properties of the modified bitumen. 

Luo and Chen [116] studied the effect of EVA (plastomer) and EVA-g-MA (reactive polymer) and found that both the polymers improved the softening point and penetration index of bitumen, but EVA-g-MA showed higher softening point temperature than EVA alone. EVA showed 14 °C, 16 °C, and 18 °C increase in softening point whereas EVA-g-MA showed 21 °C, 24 °C, and 27 °C for 4%, 5% and 6% of EVA and EVA-g-MA, respectively than that of unmodified bitumen. Similarly, the penetration index and ductility values of EVA-g-MA modified bitumen were improved compared to EVA-modified bitumen, suggesting that EVA-g-MA modified bitumen is less susceptible to temperature changes and low temperature cracking. It was concluded that a network structure is formed between EVA-g-MA and bitumen due to reactions taking place between the MA group of EVA-g-MA and functional groups of asphaltene in bitumen. This network structure helps reinforce the compatibility between bitumen and EVA-g-MA [116]. The storage stability for EVA-g-MA modified bitumen shows that the temperature difference in top and bottom samples was less than 2.5, confirming that EVA-g-MA modified bitumen is stable at higher temperature according to the storage stability cigar tube test [295]. Another study blended two reactive polymers (Elvaloy AM, containing butylacrylate 28 wt%, glycidylmethacrylate 5.3 wt% and Elvaloy 4170, containing butylacrylate 20 wt%, glycidylmethacrylate 9 wt%) with bitumen to examine the effect of these reactive polymers on storage stability. It was found that all samples exhibited complete homogeneity, suggesting that the reactive polymers guarantee better storage stability and, consequently, better bitumen-polymer compatibility [296]. Yeh et al. confirmed the better compatibility of aPP and bitumen as compared to iPP-bitumen in the presence of reactive polymers [245]. LDPE and LDPE-g-MA were blended with bitumen and it was observed that LDPE-g-MA showed greater stabilizing effect than LDPE [171]. The rheological behaviour of base bitumen and EVA-g-MA modified bitumen in terms of phase angle and complex shear modulus was investigated by [116]. It was observed that the addition of EVA-g-MA in bitumen affected the rheological properties significantly by increasing the complex modulus and reducing phase angle values. The greater the concentration of EVA-g-MA, the more significant the changes in complex shear modulus and lower phase angle. A reduction in phase angle while increasing the polymer content confirms that the elastic properties of modified bitumen have been improved, whereas an increase in complex shear modulus with increasing polymer content represents the network structure formation. 

Although polymer functionalization provides various advantages such as higher reactivity and polarity, it also comes with some drawbacks such as undesired and uncontrolled crosslinking of un-saturated polymers. Hence, it is proposed that the reactive polymers should be used with plastomers (PE and PP) due to their saturated nature [100]. Other than uncontrolled and undesired crosslinking, high cost and gelation problems are also reported as shortcomings of the use of reactive polymers. Various approaches have been suggested to overcome such shortcomings of reactive polymers. One approach is to reduce the content of reactive polymers; the majority of studies suggested using less than 1% concentration whereas very few studies proposed increasing the concentration up to 2–2.5% by weight of bitumen [66]; high loadings of such polymers form infusible and insoluble bitumen gel [67]. Bulatovic et al. [296] studied the effect of different loading percentages of reactive polymers on the properties of bitumen and found that 1.9 wt% reactive polymers resulted in gelification of the binder. Polacco et al. [297] reported that a low percentage of reactive polymers generally has an insignificant effect on the properties of the base binder. Hence, further research is required for optimizing the percentage of reactive polymer by adding co-modifiers and/or catalysts to accomplish improved properties of the binder.

### 7.2. Maleated Bitumen

**Figure 6:**
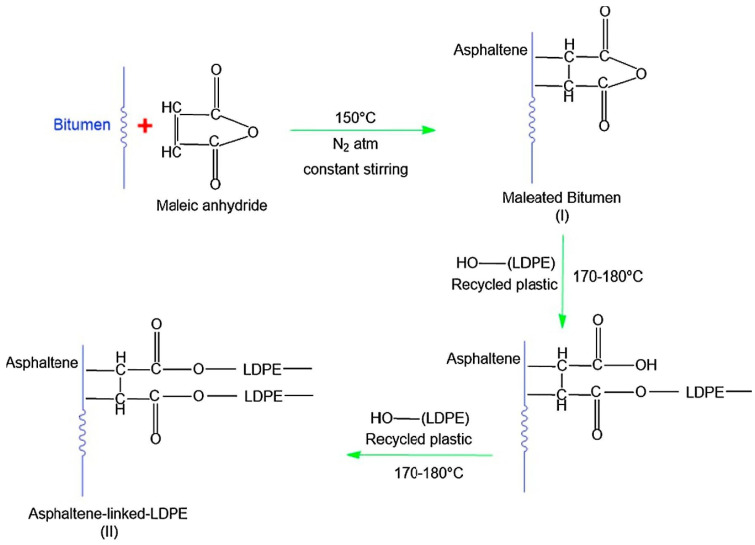
Schematic illustration of interaction occurring between LLDPE and malleated bitumen [57].

Maleic anhydride, C_4_H_2_O_3_, is a five-atom ring, cyclic, and unsaturated compound. It can be used either alone or after creating reactive polymers by adding it to polymers and finally use it for bitumen modification [100]. The chemical interaction between bitumen and maleic anhydride is a complex mechanism coming from Diels-Alder reactions or copolymerization [298,299]. Chemical reactions taking place between maleic anhydride and bitumen were analysed by FTIR and GC-MS analysis, which showed that two acid groups (i.e., HOOC-) were present, which can bond two molecules of bitumen indicating that two anhydrides undergo ring opening to donate the corresponding di-acid. Further, according to the FTIR analysis, a double bond disappeared, which showed the occurrence of chemical reaction between maleic anhydride and bitumen molecules [299]. Possible interaction between maleated bitumen and LDPE is shown in Figure 6 [57]. 

It is confirmed from various studies that maleic anhydride results in enhanced characteristics of modified bitumen due to its higher reactivity with bitumen molecules. The high reactivity of maleic anhydride will tend to increase the chemical interaction between polymer and bitumen helps to improve the overall characteristics of modified bitumen. Nadkarni et al. [300] studied the effect of different concentrations of maleic anhydride on mechanical and physical properties of modified bitumen. They observed that maleic anhydride improved the cohesive strength at high temperature and enhanced cracking resistance at low temperature. Another study investigated the effect of maleic anhydride grafted polymer (SEBS-g-MA) on the storage stability of modified bitumen and it was found that SEBS-g-MAH -modified bitumen showed better storage stability than the SEBS-modified bitumen [301]. In general, the addition of maleic anhydride improves the properties of modified bitumen significantly due to its high reactivity, which promotes the chemical interaction between bitumen and polymer. However, this high reactivity leads to issues during storage and handling. It is thus suggested to synthesise polymer-maleic anhydride compounds—namely creating a reactive polymer first—and then mix it with bitumen.

### 7.3. Polyphosphoric Acid

Polyphosphoric acid is an oligomer of phosphoric acid (H_3_PO_4_). It has achieved significant importance in the road industry for chemical modification of bitumen as a medium to improve its properties. However, phosphoric acid modification of bitumen is a complex physio-chemical process and the properties of modified bitumen blend may strongly be dependent upon the specific nature of bitumen [100]. However, it is proposed that improvements are also dependent upon the amount of polyphosphoric acid and type and concentration of the polymer [302]. The addition of polyphosphoric acid to bitumen is considered a complex process due to the large number of molecules with different structures and their possible reactions taking place during blending with bitumen. It is reported that the polyphosphoric acid tends to revert back to orthophosphoric acid after blending with bitumen [303]. According to Baumgardner et al. [304], polyphosphoric acid helps neutralize the polar interaction between asphaltene molecules of bitumen through esterification or protonation of basic sites. Other reactions which are expected to take place during mixing of polyphosphoric acid and bitumen are alkyl aromatization and co-polymerization. 

It is suggested that polyphosphoric acid plays an important role in improving the thermal, rheological, and other characteristics of modified bitumen. It has been observed that polyphosphoric acid helps improve the storage stability of polymer-modified bitumen. In terms of rheological and thermal properties, it was found that polyphosphoric acid/bitumen blends showed an enhanced high temperature performance whereas it does not significantly alter low temperature properties [305,306]. Another study used three different forms of ethylene-propylene copolymer for modification of bitumen in the presence of polyphosphoric acid and found a remarkable reduction in polymer segregation [101]. Xiao et al. [307] investigated high temperature rheological properties of polymer modified blends in absence and presence of PPA and it was found that the modified binder in presence of PPA showed the highest viscosity values. Another study evaluated the effect of PPA on EMA-GMA modification of bitumen and compared the results with SBS binder and found that EMA-GMA in presence of PPA increased stiffness as well as elasticity of binders. They claimed that PPA tends to act as a catalyst for promoting chemical reaction between bitumen and EMA-GMA and PPA enhanced the efficiency of EMA-GMA to achieve binders with similar or better performance than SBS binder [9]. PPA is a well-known alternative to be used as binder modifier although some industrial plants are reluctant on using it due its corrosive effect [284]. 

### 7.4. Sulphur

Initial applications of sulphur in bitumen were carried out alone without any other material, such as polymers. Hence, it was used in large amounts and it was suggested that the mixing temperature plays a significant role to control the reaction between bitumen and sulphur. A temperature below 140 °C was found to be the ideal temperature for bitumen and sulphur blending, because at this temperature the sulphur can easily penetrate the bitumen molecules and form hydrogen sulphide through dehydrogenation reactions [100]. This promotes reactions between the naphthenic and aromatic components, modifying the colloidal structure and chemical composition of bitumen. It is also suggested that sulphur has the potential to self-polymerize when mixed with bitumen [274]. However, polymers are nowadays added as a main modifier while sulphur is inserted in small quantity (i.e., 0.1% or less by weight of the bitumen) to help chemical reactions happen between the polymer and bitumen. The reaction of polymer/sulphur/bitumen is not fully understood, but it is assumed that the sulphur crosslinks the polymer molecules to chemically link the polymer and bitumen by sulphide and polysulfide bonds. It is also proposed that the addition of sulphur causes vulcanization of bitumen and polymer modifier, creating bitumen-polymer interconnections. These interconnections are supposed to improve the storage stability of the blends through the linking of polymer with bitumen molecules via chemical covalent bond [100]. 

Several studies have claimed that the addition of sulphur as a chemical modifier in polymer/bitumen blends improves various properties of modified bitumen including elasticity, storage stability, rheological characteristics, and resistance to deformation [307,308,309,310]. From a rheological perspective, it is proposed that sulphur-modified polymer/bitumen blends show greater elastic behaviour (lower phase angle at specific temperature and loading frequency) as compared to blends where sulphur is not added [308,311]. Although the addition of sulphur enhances different characteristics of modified bitumen, it also carries a few drawbacks while used as a chemical modifier for polymer/bitumen blends. In fact, sulphur can mainly react with unsaturated polymers due to its reactions taking place with the double bonds of polymers, commonly found in unsaturated polymers. Hence, its range of applications is limited. In addition, sulphur does not distribute throughout the mixture, hence non-homogenous polymer-modified bitumen could be obtained. Another drawback of using elemental sulphur is hydrogen sulphide (hazardous gas) emissions during the preparation of polymer/bitumen blends. A possible solution to this is through the replacement of elemental sulphur by more bitumen soluble polysulfides [312]. Poor recyclability of sulphur-modified polymer/bitumen blends is another shortcoming of the addition of sulphur into polymer-modified bitumen. Alghrafy et al. [313] studied PE modified binders with or without sulphur and found that the viscosity was increased significantly after addition of sulphur and all blends with sulphur met the Superpave viscosity requirements. They observed that sulphur improved the high temperature and aging performance of modified binders for sustainable/economical pavement construction. In another study, the effect of two cross-linking agents, i.e., PPA and sulphur was studied on storage stability and observed that both PPA and sulphur improved the storage stability and performance against fatigue of PE-modified binders [313,314].

### 7.5. Nano-Clay and Nanomaterials 

Other than functionalization of polymers, clay minerals in both nano and micro sizes are widely utilized for the modification of neat and polymer-modified bitumen binders and they improve properties such as viscosity, storage stability, stiffness, ageing, and rutting resistance [105,315]. Golestani et al. [315] studied the effect of clay minerals on properties of modified bitumen and found that the added modifiers caused an increase in resistance against rutting. Another study [316] investigated the effect of EVA, nano-clay, and EVA/nano-clay on the properties of modified bitumen and observed that all modifiers improved rheological and physical properties of the binder. Another study found that the polymer nanocomposite can improve the low temperature resistance and rutting resistance of the asphalt binder [317]. Further, both studies confirmed that the nano-composite showed better performance than individual clay mineral and other polymer modifiers. The clay localizes at interfacial polymer/bitumen regions, reducing the interfacial tension, consequently the minor phase-dispersed particles lose their typical round shape where a finer dispersion is obtained [318]. This suggests that clay plays a major role on improving the compatibility between polymer and bitumen [100]. Another study suggests that clay materials enhance the storage stability of polymer-modified bitumen by reducing the density difference between bitumen and polymer; clay also improves the resistance against ageing with barrier properties of the dispersed clay platelets [319,320]. Ouyang et al. [320] fabricated LDPE/nano-silica blends and mixed this blend with bitumen to get LDPE/silica-modified bitumen. It was found that the storage stability was increased for all percentages of nano-silica and this was justified due to clay materials reducing the density gap between asphaltene-rich bitumen phase and polymer-rich phase. The improvement in storage stability is attributed to better compatibility of LDPE and bitumen, suggesting that the clay minerals help enhance the compatibility also. A research study investigated the effect of two different clays on EVA-modified bitumen and found that nano-clay improved the thermo-mechanical properties as well as provided better homogeneity, performance, and stability [321]. In addition to that, polymer dispersion improved with the addition of nanoclay [322] depending upon the concentration and functionality of PE [323].

Together with these advantages, the use of clays also brings some shortcomings mainly due to their hydrophilic nature; therefore, it is hard to disperse small particles of clay into the polymer matrix and it commonly results in two discrete phases separation. It is confirmed that the clay resides in polymer-rich phase, indicating that storage stabilization can be achieved only with pre-formed nano-clay composites. The most commonly used clay minerals for bitumen modification are bentonite, kaolinite, montmorillonite, and organophilic montmorillonite. Among clay minerals, the montmorillonite and organophilic montmorillonite are suggested to be more effective at intermediate and high temperature.

Similar to clay minerals, the addition of any nanomaterial in bitumen—either as a modifier or as an individual component—results in reduced phase angle, increased complex shear modulus, enhanced rheological properties at both high and low temperature, and more homogenous dispersion of the polymer into the bitumen [324,325,326,327,328]. High surface area and low particle size of nanomaterials are responsible for good dispersion of the polymer within the binder, hence producing a compatible system; the reduction in nanomaterial size generally improves the mechanical performance and rheological properties of the modified blends [329]. The nanomaterials used as a modifier for polymer modification of bitumen include nano-clay, nano-calcium tri-oxocarbonate, carbon nanotube, nano-titanium di-oxide, nano-zinc oxide, and nano-silica [47,140,330,331]. Another study [332] used 1% and 2% of carbon nanotube for modification of bitumen binder and found that the modified binder showed better high temperature properties, better resistance to rutting and fatigue than the unmodified binder. It is suggested that among nanomaterial modifiers, the nano-silica and nano-titanium di-oxide have better characteristics in terms of anti-moisture performance and ultraviolet ageing [105,333]. The utilization of nanomaterials in bitumen modification has some limitations such as high cost of nanomaterials and non-homogenous mixing of nanomaterials with polymer/bitumen blends. 

### 7.6. Bio-Oil 

The bio-oils are produced by hydrothermal liquefaction or pyrolysis of biomass and are composed of complex compounds based on C, H, and O and the main constituents are alcohols, esters, ketones, aldehydes, acids, phenols, and furan [334,335]. Both hydrothermal liquefaction and pyrolysis generate solid, liquid and gases products; where the solid part is termed as biochar, gaseous product is called biogas and liquid product is known as bio-oil. Recently, bio-oil produced from pyrolysis has been proposed as a chemical modifier for bitumen binder, whereas bio-oil from hydrothermal liquefaction still needs to be fully explored as a possible modifier for bitumen.

The most commonly used bio-oils for bitumen applications are produced from pyrolysis of palm oil, soybean oil, vegetable oil, engine oil residue, microalgae, corn stover, grape residue, swine waste, and wood pellets [336,337,338]. Bio-oils have been utilized for three different purposes in the bitumen industry—i.e., 100% replacement of bitumen (bio-binder), 25–75% replacement of binder (extender), and around 10% replacement (chemical modifier) [336,339,340]. According to Kabir et al. [341] the phase separation of asphalt binders was reduced by 86% after bio-modification. 

Sun et al. [342] produced bio-bitumen, a substitute of traditional petroleum bitumen, dissolving bio-oil with styrene to get a homogenous bio-oil solution; this bio-oil solution was uniformly mixed with an accelerator (cobalt naphthenate) and an initiator (tert-butyl peroxybenzoate). The mixture was finally heated in a reactor to produce bio-bitumen. Similarly, another study proposed using switchgrass bio-oil and concluded that the rheological characteristics of the bio-binder were similar to those of bituminous binders [343]. Williams et al. [344] studied bio-oil as an extender for unmodified bitumen and polymer-modified bitumen and suggested that 9% of bio-oil addition results in significant performance improvements of bio-oil modified binders. They reported that the performance of bio-oil modifier depends upon several factors including bio-oil source, amount of bio-oil blended with binder and source and characteristics of the base binder. 

Several studies have indeed reported application of bio-oil as a chemical modifier for polymer-modified bitumen binders. It is reported that the addition of bio-oil affects physical, mechanical, rheological, and chemical characteristics of bitumen and improves swelling of the polymer-phase in the binder. Ingrassia et al. [345] added 5%, 10%, and 15% wood bio-oil by weight of bitumen and studied the chemical, morphological, and rheological properties of modified bitumen. It was found from FTIR analysis that the ester and aromatics peaks were developed after bio-oil addition. The morphological analysis confirmed that the bio-binders are completely homogenous, hence there is no risk of phase separation. They concluded that the partial replacement of binder with wood bio-oil is suitable for road applications and provides high performance against thermal and fatigue cracking resistance while also rejuvenating aged bitumen. 

The flash point and fire point are considered important tests for transportation and handling of bitumen and modified bitumen and contradictory observations have been reported in literature about the effect of bio-oil addition on the fire point and flash point of modified bitumen. Xinxin et al. [346] reported that as the bio-oil amount increases, both the fire point and flash point decreases whereas some other studies found that fire point and flash point increased when increasing the amount of bio-oil modifier [346,347]. Yang and You [348] investigated the effect of bio-oil addition on rheological properties of polymer-modified bitumen and found that an increase in bio-oil results in an increased complex shear modulus and decreased phase angle. A decrease in phase angle represents the ability of modifier to form a continuous elastic networking between the modifier and bitumen [349]. The J_nr_ value from multiple stress creep recovery (MSCR) test was decreased by increasing the amount of bio-oil, suggesting that the bio-oil possesses a potential for reducing rutting [9]. From a chemical perspective, it was observed that the addition of bio-oil resulted in a decreased intensity of carbonyl peaks (C=O), whereas the alkyl group (-CO) and alcohol stretching (-OH) appeared on bio-oil modified bitumen. These functional groups are not found in petroleum-based bitumen; hence, they are typical attributes of bio-oil [350,351]. A reduction in the intensity of carbonyl peaks is attributed to the decrease of the overall asphaltene percentage, consequently decreasing the carbonyl functional groups [352,353]. It was found that the addition of bio-oil caused a significant change in the SARA composition of bitumen, where the resin content was increased and the aromatics were decreased. The addition of bio-oil results in increasing resins and decreasing asphaltenes, saturates and aromatics [344]. A higher amount of resin increases viscosity and lowers shear susceptibility and penetration index [354]. 

**Table 5:** An overview of chemical modifiers used for improving plastomer-bitumen compatibility.

Modifiers/Compatibilizers	Method/Weight% of Modifiers	Key Findings	References
Reactive polymer (TOR)	2:1 of PE and TOR 0.1, 0.5 or 1% sulphur was mixed with PE-TOR	High stiffness with increased softening point, rotational viscosity, and decreased penetration Increase storage stability Increase elastic properties The modified binder was homogeneous, hence improved rheological properties Resistant to permanent deformation	[41]
Reactive polymer, Amorphous poly alpha olefin (APAO)	15% waste tyre rubber, 15% waste polymer and 4% APAO, 15% waste polymer and 6% APAO	APAO tends to improve anti-aging properties Better aging resistance High resistance to permanent deformation and fatigue cracking Reduced temperature susceptibility Improved high temperature performance High resistance to rutting and high storage stability Strong molecular network	[355]
EMA-GMA Terpolymer (ethylene/metilacrilate/glycidyl metacrylate) and HDPE	1.8% of EMA-GMA 0.10, 0.30, and 0.50% HDPE 0.15% and 0.30% polyphosphoric acid	EMA-GMA affected the elasticity and stiffness of binder EMA-GMA with either HDPE and polyphosphoric acid improved both stiffness and elasticity. Combined EMA-GMA, HDPE and polyphosphoric acid showed better resistance to permanent deformation	[9]
Malleated bitumen (Reaction of bitumen with maleic anhydride)	1, 2, 3, 5, and 10% of maleic anhydride was added to molten bitumen 3, 5, 7, and 9% of LDPE was added with malleated bitumen Finally, an optimized malleated bitumen/LDPE blend was mixed with 1, 2, 3, and 5% SBS, 2, 4, and 6% of natural rubber and 5 and 10% castor oil	Malleated bitumen minimizes phase separation Improved storage stability Adequate softening point and low temperature flexibility	[57]
Maleic anhydride grafted polyethylene (PE-g-MA)Maleic anhydride-grafted ethylene-octene copolymer (POE-g-MA)Maleic anhydride-graftedlinear LDPEMaleic anhydride-grafted ethylenevinyl-acetate copolymerMaleic anhydride-graftedstyrene-ethylene-butylene-styrene	The waste tyre, LDPE and compatibilizer were mixed in HAAKE rheomix at 120 C and 60 rpm for 10 min Then, this mixture was blended with 200 g of bitumen	PE-g-MA and POE-g-MA provide greater tensile stress and toughness The toughness value of POE-g-MA blend is the largest (2032.3 MJ/m^3^ compared to control 1402.9 MJ/m^3^) The swelling properties of POE-g-MA blend are the best among the five compatibilizers POE-g-MA should be a good modifier to improve the compatibility between the bitumen and the WTP/LDPE blend	[356]
LLDPE-g-MA	30%, 50% and 70% HDPE 30%, 50% and 70% CR 0%, 1%, 3% and 5% LLDPE-g-MA Initially, the HDPE/Cr/LLDPE-g-MA blends were formed by using Rheomix Then 15 wt% of HDPE/Cr/LLDPE-g-MA blend was mixed with bitumen in shear mixer	Higher content of LLDPE-g-MA shows better storage stability Less rutting and permanent deformation Good network and interaction between maleic anhydride and bitumen functional groups, therefore no phase separation at high temperatures	[357]
Electron irradiated recycled low-density polyethylene (e-LDPE_R_)	1%, 3%, 5%, 7% and 9% of e-LDPE_R_	Improved rheological properties Improved stiffness Chemical bonding of e-LDPE_R_ with bitumen Formation of mixed bitumen/e-LDPE_R_ amorphous phase	[207]
Electron irradiated recycled high-density polyethylene (e-HDPER)	1%, 3%, 5%, 7% and 9% of e-HDPE_R_	Improvement in temperature susceptibility Enhanced physical properties Chemical interaction between polymer and bitumen	[358]
Silane crosslinking agent (Si-XLPE)	0.5, 1, 2 and 3% polymer	Technical, environmental, and economical benefits More stiffness Improved storage stability, hence, better compatibility	[292]
Polyphosphoric acid	0.10, 0.30, and 0.50% HDPE 0.15% and 0.30% polyphosphoric acid	EMA-GMA with either HDPE and polyphosphoric acid improved both stiffness and elasticity Polyphosphoric acid enhanced chemical reactions Combined EMA-GMA, HDPE and polyphosphoric acid showed better resistance to permanent deformation	[9]
Sulphur	Hybrid blends of SBS with LLDPE, LDPE, EVA was prepared 0.1% sulphur was added	Sulphur offered chemical cross-linking effect It tends to help chemical bonding Complied with Superpave rutting parameter threshold by addition of sulphur	[359]
Sulphur	0.15% and 0.25% sulphur were added 4.5% and 7.5% of SBS was used as polymer modifier	Sulphur acts as a stabilising agent Sulphur cross-linking creates multi-sulfidic bond Sulphur helped with SBS polymer dispersion	[360]
Flake graphiteGraphite nanoplatelets	5% flake graphite was added The xGNP was used as 2%	Addition of graphite materials increase thermal conductivity resulting in better light healing The 5% flake graphite modified bitumen mixtures had higher healing performance than 2% graphite nanoplatelets modified bitumen	[361]
Rapeseed bio-oilFish bio-oil	Rapeseed oil and fish oil were used as chemical modifier	Both oils exhibited solubility issues Fish oil worked better than rapeseed oil for binder modification	[354]
Bio-oil from waste wood	Three types of bio-oil including original bio-oil, de-watered bio-oil and polymer modified bio-oil were used as chemical modifiers	Addition of bio-oil improved high temperature performance of bitumen binders Polymer modified bio-oil had highest stiffness followed by de-watered bio-oil and original bio-oil Original bio-oil showed the lowest effect as compared to other two bio-oils	[362]
Bio-oil	Bio-oil modified bitumen binder was prepared by adding bio-oil into bitumen binder	Thermal storage stability was decreased by increasing bio-oil content Physical segregation and chemical reactions occurred by adding bio-oil	[363]

## 8. Critical Discussion

Polymer modification is considered an effective technique to improve the bitumen properties in order to avoid or at least minimize binder failure while increasing the lifetime of the road. The three most commonly used types of polymers—chemically functionalised thermoplastics, elastomers, and plastomers—have been successfully applied for bitumen modification and each type has shown to provide various advantages and disadvantages. However, this review article focuses on the utilization of plastomers; both virgin and waste plastomeric polymers for bitumen modification. An increasing number of research studies are focusing on the application of waste plastomers for bitumen modification due to their abundant availability, easy recycling, low cost, and consistent engineering performance similar to virgin polymers. Plastomers are of various types including EVA, EBA, EMA, PP, PVC, PET, PS, and PE; however, each plastomer has its own advantages and disadvantages over others when mixed with bitumen. After reviewing a large number of studies on plastomeric modification of bitumen, it should be emphasised that some of the polymers only melt at a very high temperature, which can be higher than the bitumen production temperature. Although studies are available that mix high melting temperature plastomers with bitumen, these should be considered as inert fillers rather than active polymers.

Among the most common plastomers already used by the asphalt industry is EVA. Although this polymer is largely used in combination with bitumen and provides remarkable performance, limited studies have addressed the role of VA content on EVA-modified bitumen. Low VA content increases stiffness and the softening point, whereas it lowers penetration, tensile strength, and elongation values. The contrary is true for high VA content where the polymer behaves more like a rubber than rigid plastic. Additionally, low VA content generates stiffer bituminous blends at high temperature whereas higher VA content shows a similar behaviour to SBS modified bitumen [364]. PP-modified bitumen was found to improve rutting resistance, fatigue life, stability, and Marshall properties; although not extensively studied as polymer for bitumen, PP has demonstrated to retain interesting properties that deserve more investigation. Mainly due to environmental issues during heating at high temperature, PVC is not preferred for road applications; however, a few studies were conducted on PVC modification of bitumen and found that PVC improved physical and rheological characteristics of modified bitumen. PS modification of bitumen, similarly to the other high-melting temperature polymers, generally provided a similar stiffening effect already seen in PVC and PET; most of the studies emphasised betterments at high temperature and lack of miscibility unless modified with additives. Among all plastomers, PE is generally considered the most suitable option for bitumen modification when using recycled plastomers. Due to its low melting point temperature, it has the ability to provide homogeneous mixing with bitumen. This is demonstrated by the large number of research studies that used PE as bitumen modifier over the last two decades. In addition, PE contributes to the largest share of plastomers production worldwide, only followed by PP and PET [223]. PE has robust chemical properties and it takes longer time to degrade than others [41], hence requiring additional attention in terms of potential environmental issues [365]. 

As shown in Table 3, different percentages (loadings) of plastomers have been used for polymer modification of bitumen; however, 3–6% by weight of binder is considered as an optimal percentage for improving performance while avoiding complications during construction due to excessive viscosity and storage stability issues [192]. If 3–6% of recycled plastics is used in road construction, for instance, then up to 1.5–3 kg recycled plastomers are needed for 1 ton of asphalt, which is enough to pave 2.38 m of 1-lane road, 3.5 m wide, and 50 mm thickness of the bitumen surface layer. Hence, 630–1260 kg of plastomers can effectively be utilised for 1 km road (1 lane). These figures are significant and should carefully be considered by decision makers that foster sustainability. In fact, most of the polyolefins have already been used in the real scale by many contractors in various parts of the world. However, there are more exotic types of plastic such as PVC that are mostly studied at laboratory scale due to emissions, cost, and other problems. To promote a safe and sustainable use of recycled plastics in roads there are some issues that still deserve more investigation: 1) low temperature performance is only addressed by a very small portion of studies, 2) specific stabilisers and compatibilizers should be developed and investigated to solve storage issues, 3) life-cycle assessment studies are needed to exactly quantify the environmental savings if using recycled plastomers, 4) more focus should be put in studying the environmental aspects (i.e., fuming, emissions) of mixing recycled plastomers at high temperature. In addition, the future recyclability of plastomer-modified bitumen/asphalt is an important aspect that requires the attention of policymakers and scientists. In general, the difference in recyclability of polymer-modified bitumen and neat bitumen (if any) is still part of an ongoing research effort.

### Drawbacks and Future Works

While using waste plastic provides an additional option to redirect waste from landfills and a potential enhancement additive as a bitumen modifier, there are several concerns from an environmental perspective when introducing waste plastic on roads—one of them being the possibility of increasing fuming and emissions during bitumen production. While bitumen production is already a known contributor to VOC and PAH emissions, the introduction of waste plastics could potentially affect the total emissions produced. Therefore, more research is required in this specific field of fuming and emissions when waste plastics are introduced during bitumen production. Additionally, the possibility of microplastic release is also a common environmental concern when introducing new materials in bitumen. While there are studies concerning crumb rubber modified bitumen microplastic leaching, there is no microplastics assessment of waste plastic modified bitumen. Whilst not directly related to bitumen modification, the recyclability of plastic modified asphalt is also an area of concern. Therefore, to fully understand the effects of incorporating waste plastic in road applications, studies only involving mechanical properties are not enough to justify the addition of new foreign materials that could pose a threat in a different form (environmental). More studies related to the topics mentioned above are required before a concise decision can be made. 

## 9. Conclusions and Recommendations

The overall performance, durability characteristics as well as the failure of asphalt mixtures are highly dependent upon the characteristics of bitumen. Improved durability and performance of the binder are highly desired, hence considerable efforts are being put into polymer modification of bitumen. Plastomers are commonly used due to their relatively low cost and great stiffness, especially at high temperature. Recently, the use of recycled plastomers is also growing notable interest to possibly contribute to the broader environmental challenges posed by waste plastics.

After reviewing numerous studies, the following conclusions can be drawn.

- The incorporation of recycled LDPE in bitumen saw a decrease in penetration value (approximately 16%) at 2% polymer content—a commonly adopted polymer loading; however, increments to the softening point (approximately 15%), flash point and fire point were also noticed. Moisture resistance and bitumen’s complex modulus were also increased by 13% and 11%, respectively;
- Recycled HDPE-modified bitumen results exhibited improvements up to 89% in MSCR tests, hence emphasizing the general rheological betterment at high temperature;
- The use of commingled PE (mainly from post-consumer waste plastics) provided general benefits to the bitumen performance although more variability compared to single-source recycled plastic was noticed;
- The suggested optimum polymer content for polyethylene-based modifiers is 4% by weight of bitumen although greater polymer contents were also evaluated; the greater the polymer content, the higher the chances of phase separation during storage at high temperature;
- PET-modified binders used to make plastic-asphalt exhibited improvements in the Marshall stability by 12%. Despite improvements with the use of PET, the high melting temperature of the plastic does not allow a homogenous blend during the mixing process, therefore, making it unfeasible to be considered as a candidate for bitumen modification;
- The addition of PVC into bitumen saw a reduction in penetration values by 57% and an increment in softening point by 26%. Viscosity was increased by 300% while ductility values dipped. PVC toxicity at high temperature remains a major issue, especially when treated with phthalates of various types;
- PP-modified binders showed a reduction in penetration values by 18% to 30% at 3% polymer content and 38% to 50% at 5% polymer content. However, the softening point was improved between 4% to 30% and 11% to 43.5% at 3% and 5% polymer contents, respectively. Ductility values were reduced by 20% at 5% polymer content;
- The use of PS increased softening points by 29% and 35% for 80/100-grade bitumen and 60/70 grade bitumen, respectively, however decreasing penetration values up to 20%;
- EVA-modified binders exhibited improvements of 22% to 53% in softening point, however decreasing penetration values by 33% to 51%. The ductility of bitumen was improved by 20% at 5% polymer content. Unlike other polymers such as PP, PVC, and PE, EVA-modified binders showed no major rheological drawbacks when polymer contents were increased;
- Commingled plastics modified binders comprising of HDPE/PP exhibited an improvement of up to 179% in Marshall tests on asphalt samples;
- Plastomeric modification of bitumen is mainly achieved by the use of EVA and PE; however, more polymers (especially in their recycled form) are being experimented with in a continuous effort to find a solution to the plastics waste problem;
- Some recycled plastomeric polymers have a melting temperature which is above the bitumen mixing temperature; this implies that their use is mainly as a filler or ‘synthetic’ aggregate, depending on their size. In these cases, the cost of the filler/aggregate vs. the cost of the polymer used as ‘synthetic’ aggregate should carefully be considered as the steps involved with recycling contribute to the higher final cost;
- Low melting temperature polymers (i.e., PE, both virgin and recycled) have demonstrated their suitable use as bitumen modifiers. Recycled plastomers are also considered to be cost effective due to their lower prices in comparison to a) chemically virgin plastomers and b) commonly used elastomers. Though, when used as bitumen modifier, their relative quantity in the mix is minimal (i.e., 0.25–0.5% by weight of the asphalt mix) hence reducing the environmental benefits commonly associated with recycling;
- Generally, plastomers provide excellent high-temperature properties and relatively good—depending on the specific polymer—low-temperature behaviour (i.e., EVA at high VA content). However, most of the research studies investigating plastomers are focused on the high-temperature behaviour;
- Plastomers are also acknowledged to be prone to phase separation due to the low compatibility (molecular weight, polarity, and crystallinity) between the polymer and bitumen. However, several commonly adopted elastomers (i.e., SBS) are also not immune from separation issues. New PE-based polymers are now being tested for bitumen applications that show self-crosslinking abilities and greater compatibility with bitumen;
- To improve plastomer-bitumen compatibility, several compounds have been used. These modifiers are reactive polymers, polyphosphoric acid, organometallic compounds, sulfonic acid, silanes, maleic anhydride, carboxylic anhydride, thiourea dioxide, sulphur, antioxidants, nanomaterials, clay minerals, and bio-oils. Despite the persistent use of sulphur and PPA, new additives are being investigated by many authors with promising results (i.e., maleic anhydride to improve polarity and decrease crystallinity, or nanoparticles). The use of nano materials as stabilisers, although appealing to many, have proved to be an expensive exercise, possibly too difficult for being applied on large scale.

Finally, for what specifically concerns the recycled plastomers from waste plastics, several aspects of their inclusion within the bitumen industry still deserve deep investigation. Among these, the use of post-industrial plastics can lead to very different outcomes compared to post-consumer plastics (quality, consistency, presence of contaminants). Fuming and emissions during laboratory blending at high temperature has not been addressed yet although it may deserve a specific focus to avoid unpleasant releases of emissions at the plant and during construction. The use of waste plastomers, especially when substituting the asphalt aggregate fraction (i.e., high melting point plastics), has the potential of generating microplastics on the pavement surface due to wear and tear produced by traffic; studies on the release of microplastics should also tackle the use of waste plastics as a bitumen modifier. Some plastomers are also prone to UV degradation. Finally, the future recyclability of waste plastomer-modified bitumen mixes is also of particular interest due to the full recyclability of standard bitumen.

## Figures and Tables

**Table 2:** Basic characteristics of different plastomers including PP, EVA, EBA and PE (HDPE, LDPE, and L-LDPE).

Properties	HDPE	LDPE	LLDPE	PP	EVA	EBA	References
Density (kg/m^3^) @ ASTM 209B	938–961	890–953	917–944	820–950	920–935	930	[34,37,44,107,108,109,110,111,112,113,114,115,116,117,118]
Softening point (°C) ASTM D 1525	127	95	110–115	140–150	80–150	130	[1,37,119,120,121]
Tensile strength (MPa)	3.1–27	2.34–10.11	13–22	330–414	33	20	[107,109,111,122,123,124]
Flexural modulus (GN/m^2^) @ ASTM D790	0.307	0.203	-	-	0.02–0.17	-	[107,121,125]
Melting point (°C)	129–149	106–120	124–128	130–170	54–110	76	[39,107,108,109,110,114,116,118,126,127,128,129,130,131,132,133,134,135,136]
Thermal degradation temperature (°C)	430–480	406	424–472	410–460	290–335	315	[50,136,137,138,139]
Elongation at break (%) @ ASTM D412	500–560	300–700	650	40–350	700–1000	900	[5,107,109,110,119,122,140]
Impact strength (J)	0.941		-	-	-	-	[9]
Crystallinity (%)	52.5–86	35–47.6	48–53	-	40–65	10.6	[110,113,121,141]
Melting flow index (g/10 min) @ ASTM D1238	0.15–20	0.75–32	0.9–20	0.2–3	6	150	[5,43,110,113,119,120,132,142,143]
Chemical structure	(C_2_H_4_)_n_	(CH_2_-CH_2_)_n_	C_4_H_8_-(CH_2_-CH_2_)-C_5_H_10_	[CH_2_-CH(CH_3_)]_n_	(C_2_H_4_)_n-_(C_4_H_6_O_2_)_m_	C_9_H_10_O_3_	[144,145]

## Abbreviations

SARA	Saturate, Aromatic, Resin and Asphaltene
LCA	Life Cycle Assessment
CAGR	Compound Annual Growth Rate
PMB	Polymer Modified Bitumen
EPA	Environmental Protection Agency
PE	Polyethylene
m-PE	Metallocene-catalysed Polyethylene
LDPE	Low Density Polyethylene
VLDPE	Very Low-Density Polyethylene
LLDPE	Linear Low-Density Polyethylene
MDPE	Medium Density Polyethylene
HDPE	High Density Polyethylene
UHMWPE	Ultra-high Molecular Weight Polyethylene
PP	Polypropylene
aPP	Atactic Polypropylene
iPP	Isotactic Polypropylene
PS	Polystyrene
EPS	Expended Polystyrene
PVC	Polyvinyl Chloride
PET	Polyethylene Terephthalate
EBA	Ethylene Butyl Acrylate
EVA	Ethylene-Vinyl Acetate
SBS	Styrene-butadiene-styrene
MA-g-PE	Maleic Anhydride grafted Polyethylene
HCL	Hydrochloric Acid
PPA	Polyphosphoric Acid
XRF	X-ray fluorescence
TG/DTG	Thermogravimetric/Differential Thermogravimetric
FTIR	Fourier Transform Infra-red
GCMS	Gas Chromatography and Mass Spectroscopy
MFI	Melt Flow Index
MSCR	Multiple Stress Creep Recovery
UV	Ultraviolet
